# Current Treatments for COVID-19: Application of Supercritical Fluids in the Manufacturing of Oral and Pulmonary Formulations

**DOI:** 10.3390/pharmaceutics14112380

**Published:** 2022-11-04

**Authors:** Helga K. Ruiz, Dolores R. Serrano, Lourdes Calvo, Albertina Cabañas

**Affiliations:** 1 Department of Physical Chemistry, Complutense University of Madrid, 28040 Madrid, Spain; 2 Department of Pharmaceutics and Food Technology, Complutense University of Madrid, 28040 Madrid, Spain; 3 Department of Chemical Engineering, Complutense University of Madrid, 28040 Madrid, Spain

**Keywords:** supercritical fluids, COVID-19, micronization, bioactive components, sterilization

## Abstract

Even though more than two years have passed since the emergence of COVID-19, the research for novel or repositioned medicines from a natural source or chemically synthesized is still an unmet clinical need. In this review, the application of supercritical fluids to the development of novel or repurposed medicines for COVID-19 and their secondary bacterial complications will be discussed. We envision three main applications of the supercritical fluids in this field: (i) drug micronization, (ii) supercritical fluid extraction of bioactives and (iii) sterilization. The supercritical fluids micronization techniques can help to improve the aqueous solubility and oral bioavailability of drugs, and consequently, the need for lower doses to elicit the same pharmacological effects can result in the reduction in the dose administered and adverse effects. In addition, micronization between 1 and 5 µm can aid in the manufacturing of pulmonary formulations to target the drug directly to the lung. Supercritical fluids also have enormous potential in the extraction of natural bioactive compounds, which have shown remarkable efficacy against COVID-19. Finally, the successful application of supercritical fluids in the inactivation of viruses opens up an opportunity for their application in drug sterilization and in the healthcare field.

## 1. Introduction

More than two years have passed since the COVID-19 pandemic was declared worldwide, and more than 272 million infections have been registered around the world, with the United States, India and Brazil leading this list, and more than 5.3 million deaths being reported. The pandemic has had an enormous impact on economies, education and society in general, the latter especially due to the considerable effect on mental health due to the loss of family and friends, constant fear, social distancing and all the confinement measures implemented by the authorities [1]. 

COVID-19 has paralyzed, in addition to the health system, political and economic relations in all countries. The world economy has suffered a very large impact, which makes it impossible to calculate when it will return to pre-pandemic levels [2].

The global COVID-19 pandemic is currently ongoing with the mass vaccination campaign, which represents an important weapon to stop the pandemic. Since the start of the pandemic, the development of the COVID-19 vaccine and pharmacological treatments have exponentially grown [3]. However, vaccine administration is not enough to stop the transmission of the virus and its fatal consequences. Hence, the identification and development of better treatment alternatives are key to alleviating possible complications, such as hyperinflammation and collateral infections [4]. 

Novel drugs are under development, but the approval of new chemical entities takes at least several years until being approved by health authority bodies [5,6]. For this reason, the repurposing of drugs, such as antivirals and antiparasitic agents, used for several years to treat other diseases has become a realistic and effective strategy against COVID-19 [7]. Several clinical trials are ongoing to determine the efficacy of this virus [8].

The severe acute respiratory syndrome is a viral respiratory disease caused by a SARS-associated coronavirus. It causes generally mild symptoms very similar to those observed in common cold, including fever, cough, and shortness of breath, after about five days of a suspected infection, but in some cases, these mild symptoms can develop into severe clinical signs typical of a severe respiratory syndrome and can lead to pneumonia, multiple organ failure, severe acute respiratory syndrome, and even death. These more severe symptoms are due to the cytokine release syndrome (CRS), or “cytokine storm”, which is an overproduction of immune cells and cytokines that leads to rapid failure of the multi-organ system and tissue damage to the lungs, kidneys, and heart. Therefore, to decrease the mortality rate, one of the objectives is to develop drugs capable of modulating the immune response or suppressing the production of overactive cytokines. Mortality occurs mainly in elderly people and in patients with medical conditions that position them as high-risk patients such as high blood pressure, diabetes, cirrhosis, coronary heart disease, and patients with surgery for tumors and Parkinson’s disease [9,10,11,12].

For the diagnosis of the disease, some clinical findings can be linked to COVID-19, including a decrease in albumin, high C-reactive protein, high lactate dehydrogenase, lymphopenia, eosinopenia, high erythrocyte sedimentation rate, leukopenia or leukocytosis, hyperbilirubinemia, elevated liver enzymes and high creatinine. Images of the thorax using X-ray equipment and especially computed tomography have led to important information on the evolution of the disease. The current standard test for confirming the disease in suspected patients continues to be reverse transcription polymerase chain reaction (RT-PCR) of oropharyngeal and nasopharyngeal swabs because of it is specificity and simplicity as a qualitative assay, although false negative rates of up to 30% have been observed. The serum antibody test is another modality for the diagnosis and monitoring of SARS-CoV-2 and allows the detection of IgM and IgG antibodies, 10 and 12 days, respectively, after the onset of symptoms [10,13].

Although the future of the pandemic cannot be predicted, the fact that mutations in the virus continue to occur makes it mandatory to search for new or repositioned drugs that will help patients especially in severe cases, avoiding serious sequelae or even deaths. 

This review focuses on the drugs that are currently being administered against SARS-CoV-2 and the secondary bacterial complications of COVID-19 to propose new methodologies to improve their bioavailability. In particular, we propose the use of micronization techniques based on supercritical fluids to overcome some of the problems presented by these drugs, especially related to the high doses required, lack of targeting and their side effects. Convalescent plasma, cell therapy, monoclonal antibodies and vaccines are out of the scope of this manuscript. This review includes the application of supercritical fluids in the extraction of bioactives from natural compounds with antiviral activity to respiratory diseases and the use of supercritical CO_2_ in the virus inactivation and sterilization of personal protection materials.

A supercritical fluid (SCF) is a substance heated above its critical temperature at a pressure above its critical pressure. SCFs have properties intermediate between those of gases and liquids. They can dissolve substances like liquids but penetrate materials like gases. The density of the SCF can be also modulated with slight changes in pressure and/or temperature.

CO_2_ is the fluid most commonly used because of its mild critical temperature and pressure conditions, 31.0 °C and 7.38 MPa, respectively, making it suitable for processing heat-sensitive compounds. Among its advantages are its low cost, non-flammability, non-toxicity, recyclability, inertness, chemical stability and GRAS status. It is a gas under normal conditions, and it is eliminated after depressurization, with the possibility of being recycled, so that no solvent residues are found in the product [14].

The enormous advantages provided by the properties of supercritical fluids, especially CO_2_, have allowed their successful application in the extraction of substances from solid or semi-solid materials, such as plants, and the micronization of different active compounds by precipitation or co-precipitation, using CO_2_. In addition, due to their intrinsic biocidal and sterilizing properties, they are being used for sterilization purposes, inactivating a wide variety of microorganisms and their spores [15,16].

Supercritical CO_2_ allows the design and control of particle size depending on different operating conditions such as flow ratio, temperature, pressures, etc. [17]. In contrast to traditional micronization techniques, these techniques avoid or minimize the use of toxic organic solvents, allow much better control of the size and particle size distribution and can be implemented in continuous or semi-continuous operation mode. Furthermore, the materials prepared and used in supercritical CO_2_ are sterile, so they can be packed directly [16,18].

## 2. Viral Life Cycle and Its Relationship to Potential Drug Targets

**Figure 1:**
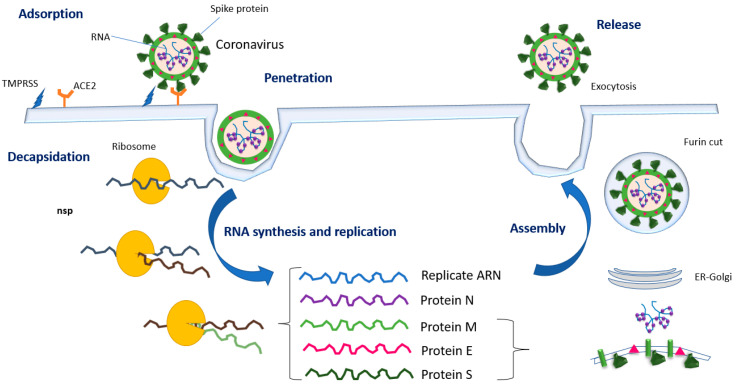
Schematic representation of the SARS-CoV-2 virus life cycle (adapted from Abu-Farha, 2020 [21]).

Human coronaviruses are characterized by presenting two groups of proteins: structural, such as Spike (S), Nucleocapsid (N), Matrix (M) and Envelope (E) [19], and non-structural proteins such as RNA-dependent RNA-polymerase (RdRp), (nsp12) and the proteases (nsp3, nsp5 or Papain-like protease, PLpro). PLpro is essential in the life cycle of viruses since it allows its replication [6,20]. Figure 1 shows the different stages of the SARS-CoV2 virus life cycle [21].

The biological cycle of the virus comprises the following steps:

- Adsorption. In this step, the virus joins the host cell, incorporating its genetic material within it. This binding is very specific and occurs through the binding of the glycoprotein S with the angiotensin-converting enzyme 2 (ACE2) receptors found on our cells in various tissues such as the oral mucosa. The S glycoprotein of SARS-CoV-2 attaches to ACE2 and human proteases including cell surface transmembrane protease/serine (TMPRSS) proteases, furin, cathepsins, plasmin, elastase, and trypsin [6,22]. Many drugs are targeting this protein to avoid the interaction with ACE2 receptors and the entrance of the virus into our cells [23].
- Penetration. Coronaviruses enter host cells through endosomal or non-endosomal pathways [24]. The COVID-19 membrane is very similar to that found in our cells, so it can easily fuse with them. Endocytosis is the second mechanism that can be used by the virus to penetrate our cells. SARS-CoV-2 entry into host cells occurs through the binding of the viral structural protein spike (S), in the receptor binding domain (RBD), to ACE2 enzyme of the host cell. When bound to the receptor, TMPRSS2 facilitates cell entry by cleaving the spike protein at the S1/S2 and S2 subunits, exposing parts that fuse the viral membrane with the receptors and host cell endosomes to enter cells; this makes TMPRSS2 or ACE2 inhibitor drug a good target for novel treatments [25,26].
- Decapsidation. The genetic material of the virus reaches the cytoplasm of the host cell. Immediately after binding, a conformational change is triggered in the S2 domain exposing the fusion peptide, mediating the virus–cell membrane fusion and eventually delivering the capsid into the cytoplasm [6].
- Synthesis and replication. Through the host cell’s ribosome, the COVID-19 RNA begins to replicate and creates multiple copies of the RNA that code for its four key proteins, S, M, N, and E. Once inside the cytoplasm and following SARS-CoV2 infection, the viral RNA unwraps and translates into polyproteins pp1a and pp1ab that encode non-structural proteins; then, continuous polypeptides are generated and cleaved into 16 nonstructural proteins (nsp1 to nsp16), which will facilitate the transcription of genomic and subgenomic RNA [6,19,27]. In this stage, the RNA-dependent RNA polymerase (RdRp) enzyme and major proteases or 3C-type proteases (Mpro or 3CLpro) are vital enzymes of the coronavirus replication/transcription and maturation and are considered attractive drug targets for the treatment of coronavirus infections [7,12].
- Assembly. These copies of RNA are transcribed into the different proteins of the virus that, within the cytoplasm of the host cell, begin to unite, forming the envelope of the virus and leaving a copy of the viral RNA inside. Viral replication organelles are generated, consisting of double-membraned perinuclear vesicles, contoured membranes and small open double-membraned spherules, creating a protective microenvironment for viral genomic RNA replication and transcription. The viral assembly occurs in the endoplasmic reticulum-Golgi compartment of the host cell, and the virions are transported to the plasma membrane via vesicles to ultimately release the mature viruses [6,19].
- Release. Through a process of exocytosis, the assembled virus copies emerge, ready to infect new cells. Virions are secreted from the infected cell by exocytosis [19].

Finally, there is a process that prepares the SARS-CoV-2 virus to infect a new cell; this step consists of a rapid five amino acid site cleavage: proline, arginine, arginine, alanine and arginine, which is essential for the attack on new lung cells. This cleavage is performed by a host cell protein called furin [26]. Coronavirus with an intact furin cleavage enters human airway cells faster than those without [28], so the furin cleavage site is also an important target for new treatments to block SARS-CoV-2 transmission [25].

## 3. Unmet Clinical Need for Treatments against COVID-19 

The pandemic COVID-19 has led to an urgent demand for the development of new potential therapeutic agents. To fight coronavirus, three strategies are devised to obtain new medicines: (i) testing existing broad-spectrum antivirals, with well-known metabolism, dosage, efficacy and side effects profile, (ii) using molecular databases and high-throughput screening to identify molecules to target specific sites of the viral cycle above described and (iii) using genomic information and pathological characteristics of different coronaviruses to develop new targeted drugs from scratch. This strategy, however, is the slowest, as the preclinical and clinical studies for new chemical entities can last more than 10 years [7].

Establishing new uses for drugs, including drugs approved or under investigation and even those that are already in disuse, alone or in combination with each other, constitutes a very efficient and low-risk promising strategy, which is due to the availability of information on the existing drug concerning pharmacokinetics, pharmacology and toxicity, among others [8,29].

At the moment, there are no registered drugs that can effectively treat SARS-CoV-2 infection [30], since those currently used are repositioned drugs from other indications for having obtained good results in in vitro studies against human coronaviruses [9]. Although there are several clinical trials registered to examine the efficacy of drugs used to treat other diseases, mostly related to viruses, there are proposed combinations of these drugs that could pose a risk to patient safety that should be further studied [8]. 

## 4. Current Drugs Used and under Evaluation for COVID-19

Several pharmacological treatments have been studied including antibiotics, anti-inflammatory/immunomodulatory agents, anticoagulants, convalescent plasma and antivirals indicated for other diseases [31]. Some studies have detected the development of resistance against antiviral therapies due to the rapid mutation rate that leads to the appearance of resistance in the viral strain. This is why sometimes, a combination of drugs has been chosen, with synergistic or additive effects, delaying the progression of the disease and increasing the survival rate of patients [32].

A brief review of the activity of current drugs used against SARS-CoV-2 is presented in Table 1, indicating the drug category, structure, activity and mechanism.

**Table 1:** Drugs used in the treatment against COVID-19 infection.

Drug	Chemical Structure	Drug Categories	Active against	Action Mechanism
Acalabrutinib	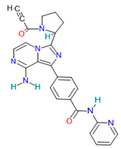	Antineoplastic agents [33]	Non-hematological disorders: glioblastoma, and carcinoma Hematological disorders: lymphoma, leukemia, and lymphoproliferative disorders [34]	Inhibitor of Bruton tyrosine kinase (BTK) [35]
Azithromycin	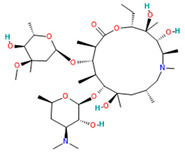	Anti-infective for systemic use [36]	Variety of community-acquired respiratory tract, skin and soft tissue, and sexually transmitted disease infections [37]	Protein synthesis inhibitor by affecting viral RNA translation [38] Immunomodulatory effect [39]
Baricitinib	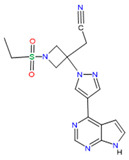	Selective immunosuppressant [36]	Rheumatoid arthritis in adult patients [31,40] Atopic dermatitis [41]	Inhibition of kinase signaling to prevent viral endocytosis. Inhibition of cytokine release by blocking the JAK1&2 [42]
Bromhexine	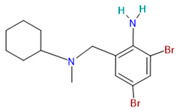	Mucolytic [36]	Used for a variety of respiratory conditions associated with increased mucus secretion [36]	TMPRSS2 inhibitor [43]
Camostat mesylate	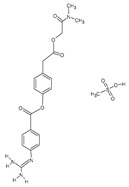	Antifibrinolytic agents [36]	Chronic pancreatitis [44] and postoperative reflux o esophagitis [45]	Inhibits TMPRSS2, then blocks nucleocapsid entry from phagosome to the cytoplasm [46]
Chloroquine and hydroxy chloroquine	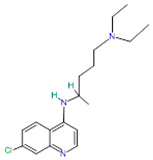	Antimalarial drugs [36]	Malaria and chronic inflammatory diseases including systemic lupus erythematosus and rheumatoid arthritis [37]	Inhibit glycosylation of host receptors, proteolytic processing, and endosomal acidification [29]
Colchicine	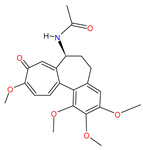	Antigout preparations [36]	Gout and familiar Mediterranean fever [47]	Host tubulin inhibitor with suppression of proinflammatory cytokines observed in people with COVID-19 [46]
Dexamethasone	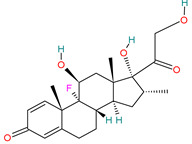	Corticosteroid [36]	Asthma, chronic obstructive lung disease, rheumatic problems, allergies, some skin diseases and also in brain edema [40,48]	Anti-inflammatory and immuno suppressive effects [42]. Inflammatory cytokine production [49]
Doxycycline	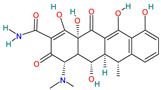	Anti-infective for systemic use [36]	Bacterial infections and a valid alternative to azithromycin [37,50]	Bacteriostatic action by inhibition of bacterial protein synthesis. Inhibitory action on metalloproteases and modulating effects of pro-inflammatory cytokines IL-6, IL-8, and TNF-α [37]
Favipiravir	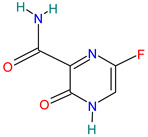	Antivirals for systemic use [36]	Arenavirus, bunyavirus, flavivirus, and filoviruses causing hemorrhagic fever and Ebola [37]	Inhibitor of RNA-dependent RNA polymerase (RdRp) [51,52]
Imatinib	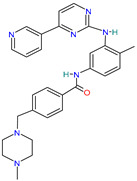	Antineoplastic and immunomodulating agent [36]	Chronic myelogenous leukemia, gastrointestinal stromal tumors and several other malignancies [36]	bcr-abl tyrosine kinase inhibitor [35]
Ivermectin	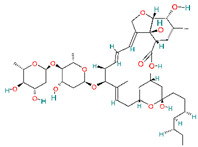	Antiparasitic agent [36]	RNA viruses such as respiratory syncytial virus (RSV), dengue, influenza, rabies, and Zika viruses [37]	Blocking the active sites of the viral protein 3CLpro and S, affecting viral replication and viral adhesion. It reduces cytokine storm [53]
Lopinavir and Ritonavir	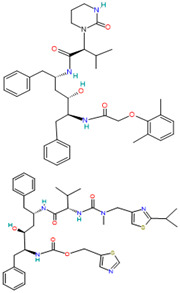	Antivirals used in combination for the treatment of HIV infections [36]	HIV, SARS, MERS [37]	3CL (Mpro) and PL protease inhibitor [29]
Melatonin	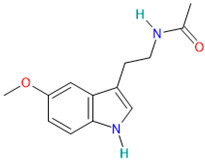	Hypnotics and Sedatives [36]	Jet lag, insomnia, shift-work disorder, circadian rhythm disorders in the blind, and benzodiazepine and nicotine withdrawal [36]	Decreases cytokines, NLRP3 inflammasomes, neutrophiles, lymphocytes and CD^8+^ -T cells [42]
Methylprednisolone	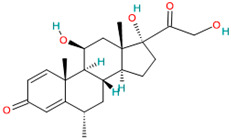	Corticosteroid [36]	Asthma and allergic rhinitis [29]	Anti-inflammatory and immunosuppressive properties [37]
Minocycline	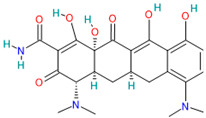	Anti-infective for systemic use [36]	Inflammatory lesions of acne vulgaris, periodontitis, and infections of susceptible microorganisms [36]	Prevent aminoacyl-tRNA from binding to the 30S ribosome, inhibiting protein synthesis [36]
Molnupiravir	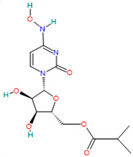	Prodrug antiviral agent [36]	COVID-19 infection [3]	Induces RNA mutations by RdRp during SARS-CoV-2 replication [54]
Niclosamide	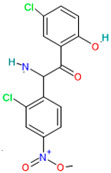	Anti-infective agent [36]	Tapeworm and intestinal fluke infections [36]	Blocks SKP2 activity, enhances autophagy and inhibits MERS-CoV replication [29]
Nitazoxanide	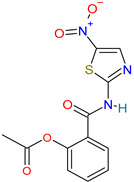	Antiparasitic agent [36]	Various helminthic, protozoal, and viral infections [29]	Inhibition expression of MERS-CoV nucleocapsid protein and suppress pro-inflammatory cytokine production, including IL-6 [55]
Paxlovid™ (Nirmatrelvir + Ritonavir)	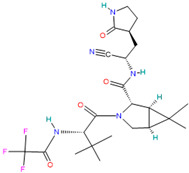	Antiviral agent [33]	Multiple human coronaviruses in vitro, including SARS-CoV, SARS-CoV-2, and other humans CoVs [56,57]	Protease (Mpro) inhibitor [56]
Remdesivir	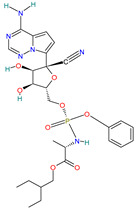	Antiviral agent [36]	Hepatitis B and C, Ebola, and Marburg virus, human immunodeficiency virus (HIV) [37]	RdRp inhibitor competing with adenosine triphosphate [46]
Ribavirin	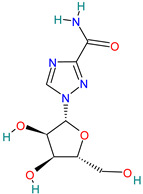	Antivirals for systemic use [36]	Human coronavirus, respiratory syncytial virus and influenza viruses [58]	Viral RdRp protein inhibitor [59]
Ruxolitinib	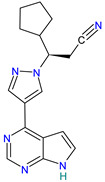	Antineoplastic and immunomodulating agent [36]	Intermediate or high-risk myelofibrosis, rheumatoid arthritis and polycythemia vera [60]	Janus kinase inhibitor [29]
Umifenovir	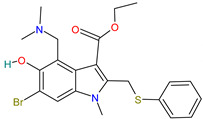	Antiviral for systemic use [36]	Influenza A and B (Russia and China) [8,38]	ACE2 inhibitor [29]

### 4.1. Approved Antiviral Therapy

The principal role of antiviral drugs is to block the viral replication cycle at any of the stages [40]. The development of an antiviral drug requires the application of strategies such as screening existing therapeutic molecule databases, the prevalence of broad-spectrum antivirals or even the synthesis of new drugs according to viral genomic characteristics [61].

Significant potential targets against SARS-CoV-2 have been identified through bioinformatics analysis, such as viral spike (S) protein, host cellular ACE2 receptor, viral genomic RNA, molecules involved in viral mRNA syntheses such as RdRp, replication complex and viral proteases, which can block different stages of the viral life cycle [6,7]. The mechanisms of action of antiviral drugs are generally to inhibit the key enzymes (such as RNA-dependent RNA polymerase, reverse transcriptase and nucleoside reverse transcriptase) of virus synthesis and consequently block viral synthesis [62]. The therapeutic potential of several antiviral drugs in the treatment of COVID-19 is currently being evaluated, mainly by co-administration with immunomodulatory drugs or antibiotics [63].

#### 4.1.1. Lopinavir (LPV)/Ritonavir (RTV)

Lopinavir/Ritonavir is a combined oral medication, which is distributed commercially as Kaletra® (400 mg LPV/100 mg RTV). It was approved by the Food and Drug Administration (FDA) in September 2000 for treating the human immunodeficiency virus (HIV), because it acts as a protease inhibitor of antiretroviral agents, such as HIV [64,65]. 

The Lopinavir/Ritonavir combination inhibits the viral enzymes 3CLpro and PLpro, which are responsible for the cleavage of the viral protein into short peptides during the assembly of new viral particles inside host cells [29]. When used together, ritonavir prevents the cytochrome P450 3A4-mediated metabolism of lopinavir to increase the drug’s bioavailability [49]. In vitro studies have been able to demonstrate that it is possible to inhibit the SARS-CoV 3CLpro enzyme [66].

#### 4.1.2. Remdesivir

Remdesivir (GS-5734, Veklury®) has been approved by the US FDA for the treatment of SARS-CoV-2 infection [67]. It is a broad-spectrum nucleoside analog, initially developed by Gilead Sciences to combat Hepatitis C [37].

Remdesivir has shown antiviral activity against many RNA viruses including Ebola, SARS-CoV and Middle East respiratory syndrome coronavirus (MERS-CoV) [6] and Marburg viruses, as well as HIV [36]. 

Remdesivir is an antiviral prodrug that is metabolized into its active form, GS-441524 [46,48]. It has been found to accelerate clinical improvement in hospitalized COVID-19 patients who do not require high-flow oxygen or mechanical ventilation [29,68]. Remdesivir is an RdRp inhibitor competing with adenosine triphosphate [46] and can inhibit the virus by inhibiting the synthesis of viral nucleic acid [7].

Remdesivir must be administered intravenously, which restricts its use at the hospital level, but it is also unstable in plasma and has a complex activation pathway that may contribute to its highly variable antiviral efficacy in SARS-CoV-2 infected cells [69]. Other routes of administration are being evaluated, and good results have been reported to date with oral [69,70] and aerosol formulations for inhalation [71,72,73], which are also prepared with precursors [74] and analogs of the drug [75].

The use of remdesivir in combination with other drugs has been evaluated with a synergistic effect. The most acclaimed combination has been corticosteroids. A possible combination of remdesivir with ribavirin, an antiviral agent with immunosuppressive effect, would enhance the suppression of the cytokine storm while inhibiting virus replication [43].

#### 4.1.3. Umifenovir

Umifenovir (registered as Arbidol) is a potent indole-based antiviral drug for seasonal influenza prophylaxis, which has been approved for the treatment of influenza A and B only in Russia and China [8,38,76].

Umifenovir has inhibitory activity against diseases such as hepatitis B and C [48]. Many studies have shown its efficacy against SARS-CoV and MERS-CoV, as it can block virus replication by inhibiting the fusion of viral particles into the target cell membrane [7] and prevents entry of the virus into host cells by endocytosis [29,37] from intracellular vesicles [77]. It affects various stages of the viral life cycle, targeting in particular virus-associated host cellular molecules or viral proteins. In vitro studies have reported the activity of the drug to interfere with vesicular attachment and trafficking in SARS-CoV-2 [6]. It also induces interferon production and exhibits broad antiviral activity [48].

#### 4.1.4. Favipiravir

Favipiravir prodrug (T-705), also marketed as “Avigan”, is a purine-based analog, which has been designed as an oral anti-influenza compound by Toyama Chemicals of Japan [38,48,51]. In 2014, it was approved for medical use in Japan for the treatment of new or re-emerging pandemic influenza virus infections [47], and in 2020 in China, for the treatment of new influenza and in the experimental treatment of emerging COVID-19 [51].

Favipiravir is a selective and potent inhibitor of RNA-dependent RNA polymerase (RdRp) of RNA viruses, preventing viral transcription and replication [51,52]. It directly targets RdRp cleavage by blocking the virion replication machinery and inhibiting infection [38,46]. Favipiravir is converted intracellularly to the triphosphate form, the active metabolite of favipiravir, and it shows broad-spectrum inhibitory activities against the RNA polymerases of influenza A viruses and many other positive-sense RNA and negative-sense RNA viruses [58]. 

#### 4.1.5. Ribavirin

Ribavirin (Virazole), is the first synthetic nucleoside analog that is active against a broad spectrum of RNA viruses (human coronavirus, respiratory syncytial virus and influenza viruses) [58]. Ribavirin is a broad-spectrum antiviral drug used to treat different viruses in combination with immunomodulators or direct-acting antivirals [78]. It inhibits the enzyme inosine monophosphate dehydrogenase and stops the replication of RNA and DNA viruses, leading to the destruction of the RNA genome [48]. 

Ribavirin is a chemical drug that inhibits the activity of viral RdRp [59] and then blocks viral replication [46]. 

### 4.2. New Antiviral Agents

#### 4.2.1. Molnupiravir (EIDD-2801, EIDD 1931-Isopropyl Ester)

Molnupiravir is an orally active and directly acting antiviral interventional drug [79]. 

It has broad-spectrum antiviral activity against multiple viruses, including SARS-CoV, MERS-CoV, and SARS-CoV-2 with a generally good safety profile [3,68]. 

Molnupiravir is the first nucleoside analog that induces RNA mutations by RdRp causing several errors during SARS-CoV-2 replication [54,80]. It is the first oral, direct-acting antiviral shown to be highly effective at reducing nasopharyngeal SARS-CoV-2 infectious virus and viral RNA and has a favorable safety and tolerability profile. It has been shown to reduce the risk of hospitalization or death in all age subgroups, with efficacy unaffected by the timing of symptom onset, underlying risk factors, or type of SARS-CoV-2 variant (gamma, delta, and mu) [3].

#### 4.2.2. Paxlovid™ (Nirmatrelvir PF-07321332 + Ritonavir)

Nirmatrelvir (PF-07321332) is the second-generation orally bioavailable SARS-CoV-2 main protease (Mpro) inhibitor [56]. 

Paxlovid has been recently announced for Pfizer [22] as a safe and effective therapy against SARS-CoV-2 in the age of Omicron variant [56], and it is recognized as a promising broad-spectrum agent. It can be used to treat infections with multiple human coronaviruses, including SARS-CoV, SARS-CoV-2, and other human CoVs [56,57].

PF-07321332 was combined for maximum anti-SARS-CoV-2 potency with ritonavir, which is an anti-HIV drug used to slow down the metabolism of PF-07321332 by inhibiting cytochrome P450 enzymes [56], and it has been shown that it significantly reduces hospital admissions and deaths among people with COVID-19 who are at high risk of severe illness [81,82].

### 4.3. Immunomodulatory Agents

Immunomodulatory drugs are being used to treat the hyperinflammatory state induced by a cytokine storm, which occurs in the most severe phases of COVID-19 infection [31,83]. As a result of SARS-CoV-2, there may be a dysregulation of the immune response leading to an exaggerated release of pro-inflammatory cytokines that induces tissue damage, causing diffuse alveolar damage, septic shock and multi-organ failure [84]. Significant inflammation in the lungs of patients is characterized by the presence and involvement of activated macrophages, neutrophils and CD4+ and CD8+ T-cells [63].

Synthetic and biological drugs, which modulate specific inflammatory pathways by inhibiting interleukines IL-6 and IL-1 or tumor necrosis factor-α (TNF-α) and intracellular enzymes Janus kinase (JAK), are used to treat inflammatory conditions [40]. Clinical trials are currently underway with several immunomodulatory therapies that regulate different aspects of inflammation for the treatment of COVID-19, including modulating the hyperinflammatory phase of the infection (dexamethasone, Janus kinase/JAK inhibitors), counteracting the cytokine storm (cytokine blocking agents), and stimulating host immunomodulatory and antiviral activity [63].

Corticosteroid therapy has had favorable responses in patients with severe COVID-19, especially in those receiving invasive mechanical ventilation [85] as demonstrated in a preliminary study evaluating the effect of dexamethasone [29]. Several studies have shown that there is a positive effect of corticosteroids in reducing immunopathological damage [83]. 

#### 4.3.1. Baricitinib

Baricitinib is an immunomodulatory drug indicated for the treatment of moderate to severe active-phase rheumatoid arthritis in adult patients [31,40]. 

Baricitinib is associated with a dual anti-SARS-CoV-2, anti-inflammatory/immunomodulatory and antiviral mechanism of action. On the one hand, it inhibits Janus kinase (JAK) 1 and 2, but it can also affect SARS-CoV-2 cell entry through its inhibitory effects on AP2-associated protein kinase, which regulates clathrin mediated virus-dependent endocytosis [29,63,84] and also stops cytokine storming [40,48].

Baricitinib combined with remdesivir is a rational treatment option in conditions where corticosteroids cannot be administered and in non-intubated patients on oxygen supplementation [29,47].

#### 4.3.2. Dexamethasone

Dexamethasone is the only immunomodulatory therapy currently widely used to treat COVID-19 [42]. Dexamethasone is a glucocorticoid with both anti-inflammatory and immunomodulatory effects [64]. 

The mechanism of action of dexamethasone strictly depends on the dose used. If a low dose is administered, dexamethasone has a genomic effect, altering genes that code for pro-inflammatory cytokines and chemokines. At a higher dose of dexamethasone, the anti-inflammatory and immunomodulatory effect results from a non-genomic pathway [40,42,86].

#### 4.3.3. Methylprednisolone

Methylprednisolone is an immunosuppressive agent and a broad-spectrum corticosteroid anti-inflammatory drug [48,49].

The efficacy of methylprednisolone against COVID-19 has been evaluated in randomized, double-blind, placebo-controlled clinical trials [47]. Methylprednisolone is primarily used to prevent damage due to excessive immune response [49]. 

#### 4.3.4. Ruxolitinib

Ruxolitinib is a potent and selective inhibitor of the protein kinases JAK1 and JAK2, mediators involved in hematopoiesis and immune function, which are involved in the transduction of other proinflammatory and anti-inflammatory cytokines [37,60,87].

Ruxolitinib is administered orally, and it has been postulated for use in reducing inflammatory cytokine storm in severely ill COVID-19 patients [60,87].

#### 4.3.5. Camostat Mesylate

Camostat mesylate is a clinically tested and repositioned serine protease inhibitor [42,65,88]. Its mechanism of action against SARS-CoV-2 is through the blockade of TMPRSS2 involved in viral fusion [42], hindering viral entry into the host cell [47,89]. It is administered orally; the most direct route of administration to inhibit SARS-CoV-2 would be priming in the respiratory tract of patients with severe COVID-19 manifestations [44].

#### 4.3.6. Niclosamide

Niclosamide is an oral antihelmintic agent with the potential to exert antiviral activity through the inhibition of oxidative phosphorylation and stimulation of mitochondrial ATPase activity [47,68]. It is effective against several viral infections with nanomolar to micromolar potency, such as SARS-CoV-2 [90], MERS-CoV, ZIKV, Hepatitis C virus and human adenovirus [91]. Oral, inhalation and injectable formulations of niclosamide are currently being studied [68].

#### 4.3.7. Colchicine

Colchicine is a low-cost, widely available drug with a known safety profile [47,92]. 

Colchicine has been proposed as a potential therapeutic for non-critical patients with COVID-19 [4], reducing mortality and hospitalization [39]. It has anti-inflammatory activity with fewer adverse effects than steroids and non-steroidal anti-inflammatory drugs [47], and it suppresses the pro-inflammatory cytokines typically seen in people with COVID-19 [46]. 

### 4.4. Antibiotics to Treat Pneumonia

During the COVID-19 epidemic, although the use of antimicrobials in viral infections is not recommended, the reuse of antimicrobial drugs has been chosen, considering the presence of pneumonia in critically ill patients [52,93]. Antibiotics are mostly used against bacterial cells and can exert a bacteriostatic effect at low concentrations or a bactericidal effect at high concentrations [50].

The combination of corticosteroids and several conventional antibiotics has been included in clinical trials for COVID-19 [94].

#### 4.4.1. Azithromycin

Azithromycin is an antibiotic belonging to the macrolide family, which inhibits bacterial protein synthesis, quorum sensing and biofilm formation [39,95]. It is used for the treatment of patients with chronic pulmonary disorders, and it acts against bacterial lipopolysaccharide (LPS)-induced inflammation in pneumonia [37]. In addition to its antimicrobial properties, azithromycin has demonstrated an immunomodulatory effect by reducing the production of pro-inflammatory cytokines and inhibiting neutrophil activation [39]. Azithromycin acts on host defense reactions in chronic inflammatory diseases, which represents a great therapeutic advantage in chronic obstructive pulmonary disease, cystic fibrosis, non-eosinophilic asthma and in the case of non-fibrotic cystic bronchiectasis [95].

In vivo and in vitro studies have shown that azithromycin has antiviral properties against rhinovirus, respiratory syncytial virus and possibly Zika virus [95], and some studies have demonstrated that the combination therapy of azithromycin and hydroxychloroquine synergistically reduced the SARS-CoV-2 viral load [96].

#### 4.4.2. Doxycycline

Doxycycline, a semi-synthetic derivative of tetracycline, is a broad-spectrum antibiotic that is considered a second-generation tetracycline and a valid alternative to azithromycin [37,50]. 

Research conducted for the treatment of COVID-19 has reported the antiviral and anti-inflammatory properties of doxycycline, with excellent results when the drug was administered alone and in combination with ivermectin [50]. However, the general use of doxycycline for the treatment of patients with COVID-19 is not recommended except under direct medical guidance and supervision [37].

#### 4.4.3. Minocycline

Minocycline is a semi-synthetic analog of tetracycline that has broad bacteriostatic activity on Gram-positive and Gram-negative bacteria as well as aerobic and anaerobic microorganisms [97,98,99]. 

Minocycline has significant potential to be repurposed as an effective therapeutic option for patients with COVID-19 in acute respiratory distress syndrome and for preventing cytokine-induced myocardial injury and multi-organ damage with consequent mortality [52,97]. Further studies are required to characterize the efficacy and safety of minocycline in patients with COVID-19 and to allow its widespread use [97].

### 4.5. Antiparasitic Drugs

#### 4.5.1. Chloroquine and Hydroxychloroquine

Chloroquine and its less toxic hydroxyl derivative, hydroxychloroquine, were discovered and used as an antimalarial agent and have been used as immunomodulatory agents to treat autoimmune conditions such as psoriasis, arthritis and lupus erythematosus [9,95]. Hydroxychloroquine has been formulated and found to be less toxic (≈40%) than chloroquine [35].

The actions of chloroquine and hydroxychloroquine differ depending on the type of microorganisms [95]. They exert an antiviral effect by mechanisms such as inhibition of entry by preventing the glycosylation of cell surface ACE2 receptors, inhibition of endosome acidification by entering the endosomes and increasing their pH to prevent RNA release and viral replication that requires low pH, increased antigen processing for major histocompatibility complex class I and II presentations enhancing the immune response, and by increasing the activity of regulatory T cells [9,48].

Chloroquine and hydroxychloroquine block sialic acid receptors, thus preventing SARS-CoV-2 from entering the upper respiratory tract [95], and they also decrease the cytokine release, mitigating the cytokine storm [9,40].

#### 4.5.2. Ivermectin

Ivermectin, a broad-spectrum antiparasitic drug with high lipid solubility, has numerous effects on parasites, nematodes, arthropods, flaviviruses, mycobacteria and mammals through a variety of mechanisms, and it also causes immunomodulation in the host [2]. 

Ivermectin has been shown to possess very good in vitro activity against SARS-CoV-2. It reduces cytokine storm and blocks the active sites of the viral protein 3CLpro and S, affecting viral replication and viral adhesion [47,53,100].

Recent research suggests that ivermectin has a prophylactic effect and would be a strong candidate for clinical trials to treat SARS-CoV-2 [53,100,101]; it is suggested that the administration of this drug may be effective in the early stages or prevention of COVID-19, but clinical trials are required to confirm this [2].

#### 4.5.3. Nitazoxanide

It is an anti-protozoal drug for treating diarrhea caused by *Cryptosporidium* spp. or *Giardia* spp. [43,102].

New studies suggest that the drug may interfere with the viral entry and intracellular proliferation of the SARS-CoV-2 virus [35]. Nitazoxanide targets numerous points of SARS-CoV-2 life cycle including an endosomal and fusional entry of the virus into the host cells [43].

Combinations of nitazoxanide with other compounds such as azithromycin, hydroxychloroquine [35], remdesivir, amodiaquine, and umifenovir exhibit significant synergy against SARS-CoV-2 [102]. Additionally, the use of this drug in the treatment of asthma offers potential benefits in treating the symptoms of COVID-19 [35].

### 4.6. Tyrosine Kinase Inhibitors

Protein kinase inhibitors possessing anticancer activity have shown broad-spectrum in vitro activity against HCV, HIV, SARS-CoV, MERS-CoV, Dengue virus (DENV) and Ebola virus (EBOV). They would block multiple pathways associated with cell differentiation and proliferation. It is expected to reduce ACE2 levels in epithelia and, as COVID-19 uses cell surface ACE2 receptors to enter the lung epithelium, its entry would be impaired, reducing the probability of infection [103].

#### 4.6.1. Imatinib

Imatinib is a bcr-abl tyrosine kinase inhibitor [35]. Imatinib protects against capillary leak and alveolar edema caused by inflammatory stimuli, and it would predominantly benefit patients with a severe course of COVID-19 [104]. 

Imatinib has also been suggested as a possible immunomodulator capable of reducing pro-inflammatory cytokines, chemokines, and vascular adhesion molecules [105]. 

#### 4.6.2. Acalabrutinib

Acalabrutinib is the inhibitor of Bruton tyrosine kinase (BTK) [35]. It is used for the treatment of B-cell malignancies such as mantle cell lymphoma and small lymphocytic lymphoma/chronic lymphocytic leukemia [34]. Acalabrutinib, as a BTK inhibitor, has shown a protective effect against lung injury by improving oxygenation, reducing dyspnea and hypoxia, and it has shown anti-inflammatory activity, reducing cytokine levels, reducing thromboinflammation and hypercoagulability, improving COVID-19 patients [34,106].

### 4.7. Alternative Drugs

#### 4.7.1. Bromhexine 

Bromhexine is a potent inhibitor of TMPRSS2 preventing the virus from entering human cells [43]. Bromhexine reduces mucus secretion by deforming secretion glycoproteins and reducing mucosal adhesions, making it easier for patients to breathe [35]. 

Bromhexine is converted in the body to ambroxol, its main active metabolite, which increases surfactant production and has antioxidant and anti-inflammatory properties. For this reason, bromhexine can be considered a candidate for the primary prevention and/or treatment of COVID-19 [107].

#### 4.7.2. Melatonin

Melatonin possesses anti-inflammatory, antioxidant, and immunomodulatory effects and preserves mitochondrial function during conditions of oxidative stress [42], thereby exerting an indirect antiviral action [89,108].

Several studies have found that melatonin exerts other important supportive effects in the fight against COVID-19, including preventive effects against sepsis-induced renal injury, cardiomyopathy and liver injury. In addition, it exerts neurological protection by reducing the cerebral inflammatory response, cerebral edema and blood–brain barrier permeability, reducing sedation use and the frequency of pain, agitation, and anxiety, and it improves sleep quality in patients in the intensive care unit (ICU) [108]. The administration of melatonin in COVID-19 patients would therefore reduce the cytokine storm and the generation of free radicals [42]. 

**Figure 2:**
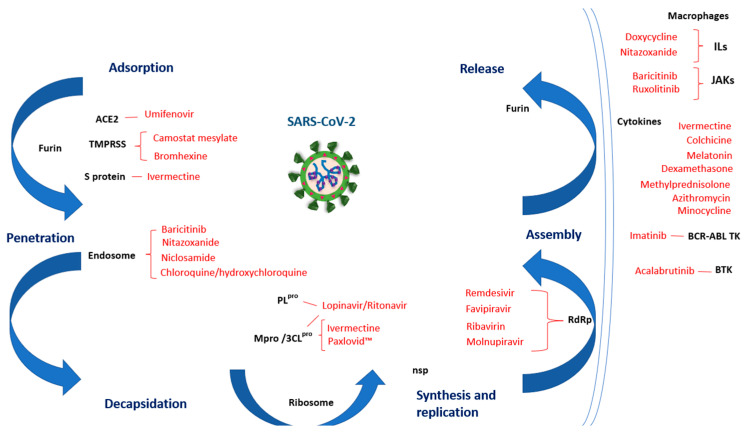
Drugs and their target within the different stages of the SARS-CoV-2 viral cycle.

Figure 2 shows the targets on which the different drugs against SARS-CoV-2 act within its life cycle and can control the inflammatory effect associated with the disease.

### 4.8. Natural Products

There are numerous publications related to the use of plants and phytochemical compounds as important allies in the fight against COVID-19. Studies have confirmed the benefits that plants provide against respiratory viruses when used as raw extracts or directly using their active ingredients in their pure form [109]. Among the natural components identified for their effect against the COVID-19 virus are, firstly, terpenoids that play a vital role in modulating cellular metabolism and that have antiviral activity, secondly, dipeptides, which act as inhibitors of protease, thirdly, sulfated polysaccharides, which have been recognized for their antitumor, anticoagulant, antiviral and immunomodulatory activity, and finally, polyphenols, which have been identified as potent COVID-19 protease (Mpro) inhibitors [110]. 

Among polyphenols, flavonoids are the most studied components against coronavirus [111,112]. Quercetin is one of the most abundant plant flavonoids, being the leading candidate for its ability to inhibit the interaction between SARS-CoV spike protein and ACE2, viral protease and helicase activities as well as inhibiting host cell ACE2 activity and increasing the level of intracellular zinc [111]. It has also been shown that quercetin administered orally, in combination with antiviral drugs, can reduce the recovery time of COVID-19 patients [113]. It has been suggested that the administration of a nasal spray of quercetin diluted in a suitable vehicle during the early stages of infection could attenuate the entry of the virus into the cells and delay the progress of the infection, thus avoiding the need for hospitalization [114].

Other flavonoids that are widely used in the formulation of many herbal drugs are hesperidin and hesperitin as anticancer and antioxidant agents, and promising evidence has been recently reported for the prevention and treatment of COVID-19 using these compounds [115].

Some important plants such as *Artemisia annua L*, *Isatis tinctoria L*, *Lindera aggregata*, *Pelargonium sidoides DC.*, and *Glychirrhiza* spp. have been used against SARS-CoV, and other active ingredients isolated from them, such as emodin, reserpine, aescin, myricetin, scutellarin, apigenin, lutein, and betulonic acid, have shown promising results against coronaviruses [109].

In addition, bee products such as honey, pollen, propolis, royal jelly, beeswax and bee venom, which have mixtures of potentially active chemicals, have demonstrated potent antiviral activity against pathogens causing severe respiratory syndromes, including those caused by human coronaviruses, with benefits on the immune system, such as the induction of antibody production, maturation of immune cells and stimulation of innate and adaptive immune responses [116,117].

Although the use of alternative drugs and natural products in complementary or alternative therapies to fight COVID-19 may provoke some skepticism among the medical community, it is clear that their use alone or in combination with other drugs presented in Table 1 and Table 2 may help to alleviate the symptoms and possible complications derived from this disease.

## 5. Pharmacokinetic Challenges to Overcome with Current COVID-19 Treatments

Currently, new and registered drugs, alone or in combination, are still being investigated to effectively treat coronavirus infection. The current treatments either as a single drug or in combination may be deficient in terms of pharmacokinetics performance and present a safety risk [8].

Based on the above-mentioned treatments, it is a clear fact that many of them are repurposed drugs and hence, their pharmacokinetic profile is not adapted necessarily to COVID-19. For example, inhaled drugs can target better and more efficiently the drug to the lungs limiting the exposure to other tissues and reducing side effects. In Table 2, the administration route, the solubility, bioavailability and side effects of the current treatments against COVID-19 are summarized. 

**Table 2:** Drugs against COVID-19 infection, characteristics related to solubility, administration route, bioavailability and toxicities/adverse effects.

Drug	Solubility	Administration Route	Bioavailability	Toxicities/Adverse Effects
Acalabrutinib	Freely soluble in water at pH values below 3 but is practically insoluble in water at pH values above 6 [33]	Oral: capsules [33]	25% [36]	Serious and opportunistic infections, hemorrhage and cytopenia [35]
Azithromycin	Soluble in ethanol and DMSO, minimally soluble in water [33]	Oral: hard capsules, tablets, powder for oral suspension. Solution drops ophthalmic Injection [33]	37% [33]	Abnormal heart rhythms, liver problems, myasthenia gravis [38] Sudden cardiac failure, cardiovascular death, heart dysfunction, and inflammation or laryngitis [59]
Baricitinib	Water solubility: 0.357 mg/mL [36]	Oral: suspension, tablet [33]	Approximately 79% [33]	Decreased neutrophils as well as lymphocyte counts [59] Hyperlipidemia and increased risk of thromboembolism [63]
Bromhexine	Water solubility 0.01 mol/L [33]	Oral: tablets, syrup, elixir [36]	22–27% [36]	Nausea, rash, vomiting, diarrhea, allergic reactions [107]
Camostat mesylate	Water solubility 0.0626 mg/mL [36]	For oral administration, but under study as inhalation [44]	Data not available	Rash, pruritus, nausea, abnormal laboratory test values, and diarrhea [36]
Chloroquine	Very slightly soluble in water [33]	Oral: tablets, solution Injection [33]	Solution 52–102% and tablets 67–114% [36]	Arrhythmias, heart failure, ventricular hypertrophy, hypokinesia, valvular dysfunction, pulmonary hypertension [59]. Diarrhea, alopecia, and loss of appetite [38]
Hydroxychloroquine	Water solubility 0.0261 mg/mL [36]	Oral: tablets [33]	67–74% [36]	QTc prolongation, abdominal pain, decreased appetite, diarrhea, nausea, vomiting, hemolysis, hypoglycemia, retinopathy, nervous system disorder and psychiatric disorder [87]
Colchicine	1 g dissolves in 22 mL water [33]	Oral: tablets, capsules and solution [33]	About 45%, according to the FDA label [36]	Gastrointestinal tract adverse effects are the most frequent. Myelosuppression, leukopenia, granulocytopenia, thrombocytopenia, pancytopenia, and aplastic anemia [33]
Dexamethasone	Solubility in water (25 °C): 10 mg/100 mL [33]	Oral: solution, tablets, powder Injectable solution [33]	70–78% [33]	Stomach pain or cramp, headaches, dizziness, insomnia, menstrual cycle changes, and weight gain, hyperglycemia, gastrointestinal hemorrhage, and psychosis [59]
Doxycycline	Very slightly soluble in water [36]	Oral: tablets, capsules, suspension Injection [33]	90%–100% [99]	Esophagitis, esophageal ulceration, and mediastinitis [50]
Favipiravir	Slightly soluble in water [33]	Oral: tablets [36]	Almost complete 97.6%. [36]	Weight loss and increased serum concentration of liver function enzymes [59] Teratogenic [33]
Imatinib	Very soluble in water at pH < 5.5 (mesylate salt) [33]	Oral: capsules [33]	98% [33]	Edema, nausea, vomiting, muscle cramps, musculoskeletal pain, diarrhea, rash, fatigue and abdominal pain [33]
Ivermectin	Poor solubility in water [118]	Oral: tablets Topical: cream, lotion [36]	Data not available	Rash, edema, headache, dizziness, asthenia, nausea, vomiting, and diarrhea [36]
Lopinavir and Ritonavir	Lopinavir: Practically insoluble in water. [33] Ritonavir: Water solubility: 0.00126 mg/mL [36]	Oral: solution and tablets [33]	Lopinavir alone has low oral bioavailability (≈25%), it is co-administered with ritonavir, which improves its bioavailability. The absolute bioavailability of ritonavir has not been determined [33]	Nausea, diarrhea, vomiting, elevated transaminase and lactate levels, icterus and dyslipidemia. Asthenia, abdominal pain, headache, rash. Increased in total cholesterol and triglyceride concentrations [8]
Melatonin	Water solubility: 0.143 mg/mL [36]	Oral: tablets [36]	The absorption and bioavailability of melatonin varies widely [36]	Occasional dizziness, headache, nausea and sleepiness [108]
Methylprednisolone	Practically insoluble in water [33]	Oral: tablets. Parenteral: injection [33]	Oral methylprednisolone has 89.9% of the bioavailability of oral methylprednisolone acetate, while rectal methylprednisolone has 14.2% of the bioavailability [33]	Change in heart rate, lungs, chest or respiratory depression. Increased heart rate. Arrhythmias [33]
Minocycline	Water solubility: 3.07 mg/mL [36]	Oral: capsules Topical and intravenous Subgingival microspheres [36]	90%–100% oral [99]	Nausea, vomiting, headache, dizziness, discoloration of the skin and teeth, phototoxicity, susceptibility to superinfections. Potential teratogenicity risk [97]
Molnupiravir	Water solubility: 5.77 mg/mL [36]	Oral: tablets [3] Oral: hard capsules [33]	Data not available	Headache, nausea, and diarrhea. Influenza- like syndrome, back pain, rhinorrhea, hot flashes, and pain in the extremity [80]
Niclosamide	Almost insoluble in water [33]	Oral: tablets Powder for sprays (70%) and as an emulsion concentrate (25%) [33] Oral, inhalation and injectable formulations are currently being studied [68,119]	10% oral [91]	Nausea and vomiting, abdominal discomfort including anorexia, diarrhea, drowsiness, and dizziness [33]
Nitazoxanide	Poor solubility in water 0.0076 g/L [33]	Oral: tablets and suspension [102]	Suspension is 70% [36]	Abdominal pain, nausea, diarrhea, headache, and changes in urine [59]
Paxlovid™ (Nirmatrelvir + Ritonavir)	Nirmatrelvir: Water solubility: 0.0277 mg/mL [36]	Oral: tablets [36]	Data not available	Dysgeusia (taste disturbance), diarrhea and vomiting [120]
Remdesivir	Poorly soluble in water (0.028 mg/mL) [121]	Intravenous: Powder for concentrate for solution for infusion. In evaluation: Oral and inhalation (aerosol formulation) [69,71,72,73]	Data not available	Elevation of hepatic enzymes, hypotension, renal impairment, rash, diarrhea. Acute kidney injury, hypotension, septic shock and multiple organ dysfunction syndrome [8]
Ribavirin	Water-soluble Slightly soluble in alcohol [33]	Oral: solution, hard capsules and tablets [33]	64% following a single oral dose administration of 600 mg ribavirin [33]	Hypocalcemia, hemolytic anemia and hypomagnesemia [6] Nausea, weight loss or gain, and diarrhea [59]
Ruxolitinib	Soluble in buffers across a pH of 1–8, in water, 100.8 mg/L at 25 °C [33]	Oral: tablets [33]	About 80% [33]	Thrombocytopenia, neutropenia, anemia, infections, edema, headache, dizziness [87]
Umifenovir	Water solubility: 0.00678 mg/mL [36]	Oral: tablets or capsules [76]	40% [77]	Acute allergic reaction, nausea/vomiting [59]. Mainly gastrointestinal and increased transaminase levels [8]

Orally administered medicines require possessing adequate solubility and sufficient intestinal absorption to reach the sites of action and exert their therapeutic effects [62]. Otherwise, the overall performance will be hindered. 

A common problem with antiviral drugs is that they are poorly soluble in water or physiologically buffered saline and are often poorly absorbed in the gastrointestinal tract [122]. On the other hand, many immunomodulatory agents have toxicity problems, as well as solubility, stability and bioavailability limitations, resulting in a high degree of pharmacokinetic and individual variability, which ultimately causes a lack of efficacy due to the presence of subtherapeutic concentrations [63]. Antiparasitic drugs, such as ivermectin, niclosamine, and nitazoxanide, also have poor water solubility, which translates into low bioavailability for oral administration. Thus, their adverse effects should be taken into consideration due to their impact on patients.

Numerous advanced pharmaceutical techniques have now been applied to increase the solubility and permeability of antiviral drugs, including the addition of auxiliary ingredients, such as surfactants, and the application of new preparation methods, such as inclusion technology, solid dispersion technology, micronization and nanotechnology [62].

Considering that the bioavailability of the pharmaceuticals presented in a solid formulation is highly dependent on particle size, particle size distribution and particle morphology [123], the micronization process emerges as a necessary alternative to improve the drug properties, including solubility, and reducing side effects.

## 6. Need for Novel Formulations: Pulmonary Delivery

Although oral administration still constitutes the main delivery route for pharmaceutical drugs [124], since the respiratory tract is often the portal of entry for viruses causing respiratory infections, the development of inhaled drugs is of great interest. Both viral and bacterial respiratory infections usually start in the nasopharynx and/or parts of the bronchial tree, with disease worsening as the pathogens and associated inflammation migrate to the lower parts of the respiratory system. In fact, the primary site of infections of SARS-CoV-2 is the lower respiratory tract. The virus has a tropism for alveolar macrophages and type I and II pneumocytes, causing respiratory distress, and pneumonia among other complications. Thus, it is rational to administer drugs, such as antivirals, by inhalation to maximize the concentration of the drug in the lung limiting their exposure to other tissue and hence, reducing toxicity [119,125].

On the other hand, pulmonary administration is an efficient method for delivering antibiotics directly to the lung, with three types of inhaled formulations pressurized, nebulized and dry powder. The direct action of the drug in the lungs enhances the antimicrobial effect and leads to a reduction in the dose. Then, the purulence of the disease and its possible severe complications are reduced with a low systemic exposure avoiding at the same time the systemic side effects [126,127,128].

The pulmonary administration of dry powder formulations has several advantages, such as good stability and easy administration, being able to administer high doses of drugs, which in the case of antimicrobials is essential for the treatment of pulmonary infections [125].

The dose of the drug deposited in the lungs and its distribution will determine the therapeutic effect, but this distribution will depend in turn on the drug particle size, the inhalation device and its use according to the breathing pattern and the lung anatomy of the patient [127]. The recommended aerodynamical particle size for administration by inhalation is between 1 and 5 μm. Smaller sizes have a greater tendency to be eliminated by exhalation, and larger sizes above 5 μm are less likely to reach the lung parenchyma [119,124,127].

Amongst all drugs described in Table 2, none are commercialized by pulmonary administration, and only two are under investigation such as niclosamide [68,119] and camostat mesylate [44].

## 7. Use of Supercritical Fluids in the Fight against COVID-19

The use of supercritical fluids in pharmaceutical applications has increased in recent years due to their great versatility. These applications include micronization, crystallization, micro- and nano-encapsulation, controlled particle deposition, sterilization, coating processes, the formation of solid dispersions and complexes, and the generation of biopolymeric sponges or microporous foam [18,129,130,131,132]

In the context of the present revision, we believe that the use of supercritical fluids can be very beneficial in the fight against COVID-19. We envision three different fields of action: (i) the micronization of the drugs for pulmonary delivery or to improve their oral pharmacokinetic profile, (ii) the supercritical fluid extraction of bioactives from natural compounds with antiviral activity, and (iii) the use of scCO_2_ in the virus inactivation and sterilization of contaminated material.

### 7.1. Micronization of Drugs Using Supercritical Fluids

The micronization of drugs allows modifying their pharmacokinetic and pharmacodynamic properties by improving their bioavailability, their solubility and consequently reducing their adverse effects. The application of micronization techniques based on the use of supercritical fluids emerges as a viable and user-friendly environment alternative to more traditional techniques. There are excellent reviews and monographs covering the different supercritical micronization techniques and their applications [15,123,133,134,135,136,137,138,139]. Here, we will review them concisely to discuss their possible use in the preparation of COVID-19 medicines.

Conventional processes for generating micronized particles have some limitations, including the possibility of polymorphic transitions, inapplicability to thermolabile or waxy materials, low batch-to-batch reproducibility, complex scale-up, a wide range of variation in particle size distribution and the presence of residual solvents [140].

In contrast, the precipitation of pharmaceutical products and natural substances using supercritical fluids presents several advantages over conventional methods. These methods lead to particles of small sizes, being possible to control the morphology and particle size distribution [123,141]. This is because these characteristics are related to the properties of SCF that can be adjusted with small changes in pressure and temperature [123], which allows the preparation of microparticles, sub-micro and nanoparticles in a controlled manner. The temperature used in these processes is low to moderate, so the degradation of the product is much prevented. Furthermore, the use of toxic organic solvents is avoided or reduced, being the residue in the resulting product easily eliminated [141]. Reduction in the drug particle size improves its dissolution rate and bioavailability and allows lowering the drug dose and, at the same time, reducing its side effects [142]. The application of supercritical fluids is not only being applied for pulmonary administration, but it is also being used to improve the dissolution of poorly soluble compounds for oral administration [18].

According to its solvating behavior, these processes can be classified into different groups depending on the scCO_2_ processing role: acting as a solvent, cosolvent, solute, antisolvent, or as cosolute [137,139]. The most common micronization techniques are reviewed in the next section.

#### 7.1.1. Supercritical CO_2_ as a Solvent

##### Rapid Expansion of Supercritical Solutions (RESS) 

**Figure 3:**
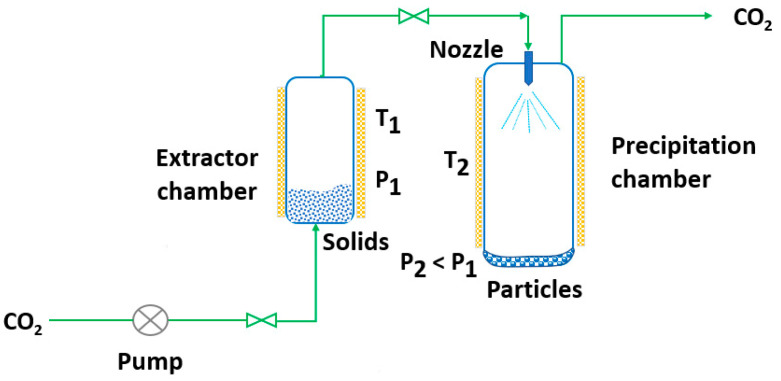
The rapid expansion of supercritical solvent (RESS) process (adapted from Parhi and Suresh [145]).

RESS was patented in 1986 by Smith and Wash [143] after the report of Kukronis [144]. This technique is based on the ability of CO_2_ to dissolve solids. The process is divided into two steps. In the first step, the compound of interest (solute) is dissolved in the supercritical fluid until saturation. The mixture of the solute and the supercritical fluid is then depressurized in the second step in a precipitation chamber through a nozzle. This creates a decrease in the SCF solvation power, leading to supersaturation and causing the precipitation of the solute [123] (see Figure 3). The temperature of the extractor/saturator chamber, nozzle and expansion chamber must be carefully controlled.

RESS has been applied to the micronization of poorly water-soluble compounds, increasing the drug dissolution rate [137,141,146,147]. Particles precipitated by RESS have particle diameters in the sub-micron range [147]. The main advantages are that it is a simple technique and it minimizes the use of organic solvents [145]. In a continuous process; the SCF is always reused. This process is only applicable to substances with high solubility in the selected supercritical fluid, for CO_2_ mainly non-polar or volatile polar compounds, and it requires the use of relatively high pressures [123,141]. The low solubility of drugs and polymers in supercritical CO_2_ makes the particle recovery difficult, leading to scale-up limitations [137]. To overcome these problems, modifications to the RESS technique have been proposed, resulting in RESS-SC [148,149] and RESOLV [150]. In RESS-SC, additives are used to improve the solubility of the compound to micronize in CO_2_, whilst in RESOLV, the product is expanded into a liquid solvent. Thus, the main advantage of this technique, related to the absence of solvents in the final product is lost, and additional stages to remove the solvents from the product are required [141].

##### RESS with Solid Cosolvent (RESS-SC)

In RESS-SC, a solid cosolvent such as menthol is added to improve the solubilization of the solute in the supercritical fluid and to provide a barrier for the coagulation of the particles leading to much smaller particles than the conventional RESS technique [148]. The mixture is expanded through the nozzle into the expansion vessel where it precipitates. Then, the solid cosolvent is removed from the precipitate by sublimation [145].

##### RESS into a Liquid Solvent (RESOLV)

RESOLV involves the rapid expansion of a supercritical solution into a liquid solvent. This allows obtaining particles with a diameter less than 50 nm [137,151]. The advantage of this method is the possibility of avoiding particle growth in the precipitator [145]. This technique may require an additional stage to remove the solvent from the product [141].

#### 7.1.2. Supercritical CO_2_ as Solute

##### Particles from Gas Saturated Solution (PGSS)

This technique was first proposed by Weidner et al. in 1995 [152]. Since then, it has been widely used in the pharmaceutical field to micronize different compounds [137]. In PGSS, CO_2_ dissolves into the substance to micronize but the system does not have to be fully miscible. Thus, PGSS can be applied to a much wider variety of substances [141]. 

**Figure 4:**
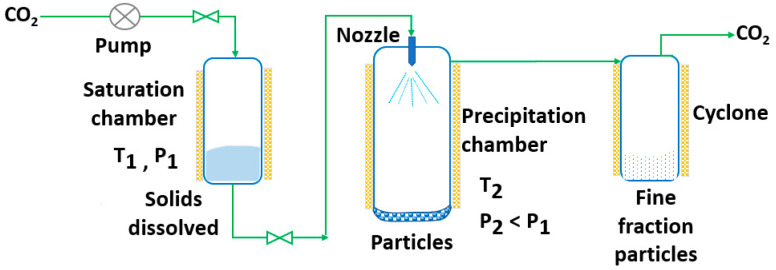
Particles from gas-saturated solutions (PGSS) process (adapted from Martin and Cocero [123]).

In this technique, CO_2_ is fed into a saturation chamber and is dissolved in a melted substance at high pressure, generating a gas-saturated solution with reduced viscosity. Then, this solution is rapidly expanded through a nozzle to a precipitation chamber at lower pressure. Upon expansion, droplets are formed and CO_2_ evaporates, leading to supersaturation, solidification and particle precipitation (see Figure 4). The driving force for particle formation in PGSS is the large temperature decrease during expansion due to the Joule–Thompson effect [153].

This technique was first applied to the micronization of different molecular weight polyethylene glycols (PEGs) [154]. PGSS is easily scalable, requires no organic solvents, and can take place at low temperatures. Furthermore, it is a simple process, which leads to a low processing cost and a wide range of applications [137,145]. 

PGSS has also been adapted to encapsulate bioactive molecules, such as Vitamin B_2_, in solid lipid nanoparticles [137]. This process can be also operated in a continuous mode, especially suitable for processing polymers and lipids in which CO_2_ has a large solubility [139,153].

As a disadvantage, the technique is limited to pharmaceutical compounds in which CO_2_ is highly soluble. Moreover, if the solute must be melted, that represents a potential issue for heat-sensitive materials [137]. Thus, the PGSS technique has been implemented successfully at the industrial level mainly in food applications [153].

In order to expand its applications and/or overcome the main limitations of the PGSS process, several modifications were applied, including emerging Concentrated Powder Form (CPF) [155,156] and Continuous Powder Coating Spraying Process (CPCSP) techniques [157]. 

##### Concentrated Powder Form (CPF)

**Figure 5:**
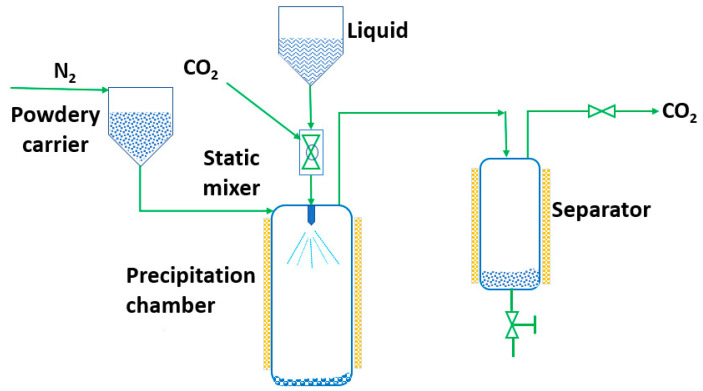
Concentrate Powder Form (CPF) process (adapted from Vorobei and Parenago [141]).

In CPF, a liquid compound and a supercritical fluid are mixed in a static mixer, and the resulting solution is sprayed through a nozzle into a precipitator chamber at low pressure. A dispersed spray of fine liquid droplets is formed during the expansion step of the gas-saturated liquid instead of solid particles. Then, an inert gas (e.g., N_2_) along with a carrier in powder form is blown into the precipitation chamber. The liquid dispersed in the form of very small droplets mixes with the carrier, forming solid agglomerates [133,139,141,155,156]. CPF allows obtaining liquid-filled powders with concentrations of up to 80–90 wt % of the liquid [139,141,153] (see Figure 5). 

##### Continuous Powder Coating Spraying Process (CPCSP)

**Figure 6:**
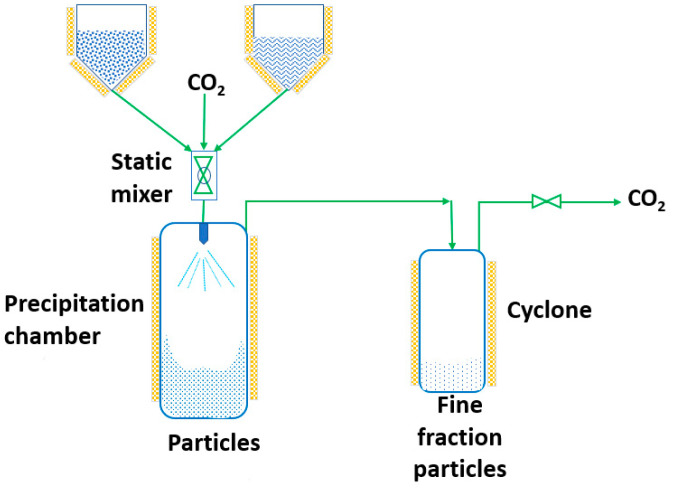
Continuous Powder Coating Spraying Process (CPCSP) (adapted from Nunes and Duarte [139]).

CPCSP is a modification of the PGSS process that was proposed as an alternative technique for the manufacture of powder coatings. It is very effective in low melting point polyester powder coatings, achieving an average particle size of less than 40 µm [157] (see Figure 6).

In this technique, each component of a powder coating mixture, the binder and hardener, are melted in separated high-pressure vessels, and high-pressure pumps fed both streams to a static mixer where the mixture is homogenized with compressed CO_2_. The solution is expanded through a nozzle into the precipitation chamber, and solid particles are formed. Finally, these particles can be separated from the gas using a cyclone separator and a filter, achieving a fine powder coating [133,139]. 

#### 7.1.3. Supercritical CO_2_ as Cosolvent 

##### Depressurization of an Expanded Liquid Organic Solution (DELOS)

This process has been developed and patented by Ventosa et al. [158,159]. It allows the preparation of micron and submicron crystalline particles of high polymorphic purity and morphological homogeneity, free of residual solvent in one step [133,137,140]. DELOS can be also used to produce polymorphs that cannot be obtained with other crystallization techniques [159].

**Figure 7:**
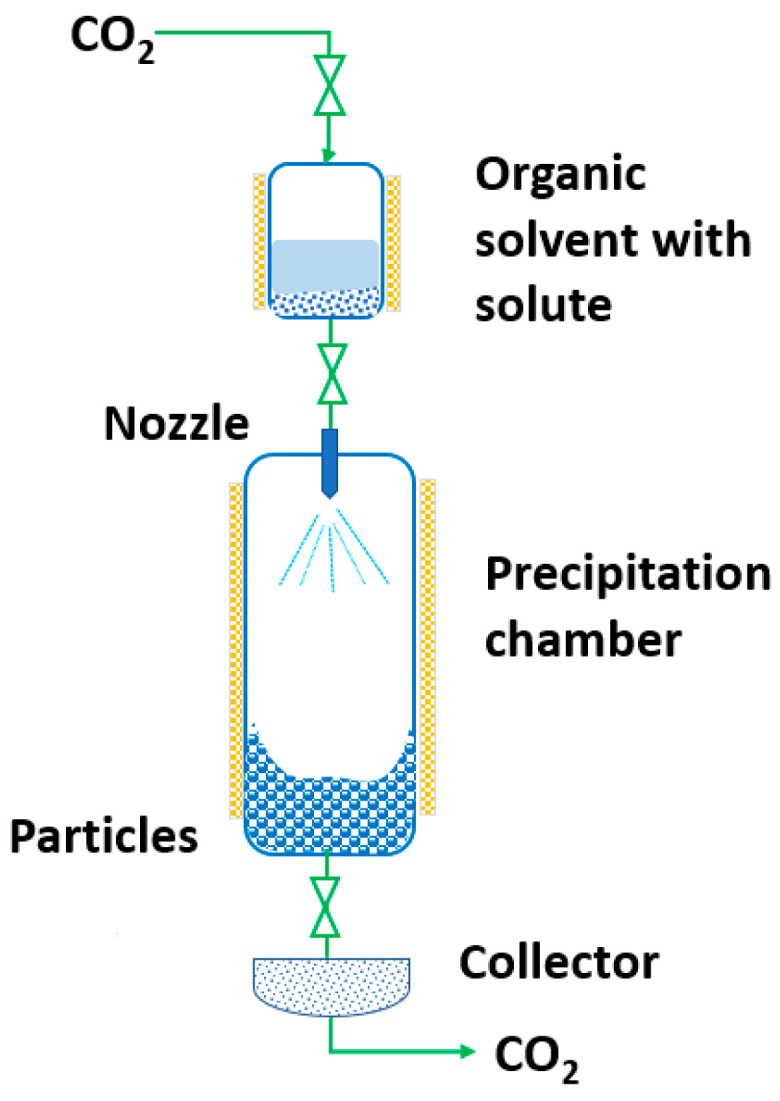
Depressurization of an Expanded Liquid Organic Solution (DELOS) process (adapted from Costa et al. [137]).

In DELOS, scCO_2_ is used as a cosolvent to saturate an organic solution of the solute of interest, forming a volumetric gas expanded liquid solution, which is then expanded through a nozzle [123,139,145] (see Figure 7). Upon expansion, CO_2_ evaporates, the solution then becomes supersaturated, and the solid precipitates. Due to CO_2_ evaporation, the system cools rapidly and homogenously, leading to crystalline materials. This process can only be applied to substances for which CO_2_ does not have an antisolvent effect, as otherwise, the solute precipitates in the saturator and not in the precipitation chamber [158,160].

The main advantage of this technique in comparison to PGSS is that thermo-sensitive materials can be handled to prepare fine particles without melting [145]. A modification to the DELOS process, named DELOS-SUSP, consists of depressurizing the gas-expanded liquid solution into another solvent that interrupts the crystallization [161,162]. This process is being applied on an industrial scale [15,139].

#### 7.1.4. Supercritical CO_2_ as Cosolute

##### Carbon Dioxide-Assisted Nebulization with a Bubble Dryer (CAN-BD)

This technique enables the micronization of water-soluble compounds such as inorganic salts or proteins [137]. It was first proposed by Siever et al. [163,164].

**Figure 8:**
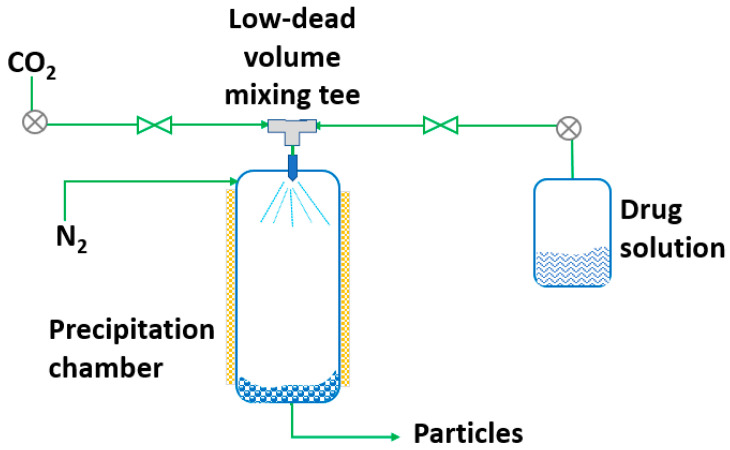
CO_2_-Assisted Nebulization with a Bubble Dryer (CAN-BD) process (adapted from Costa et al. [137]).

In the CAN-BD process, the drug is dissolved or suspended in water or an organic solvent (or both), preferably in a concentration between 1% and 10% of the total solids dissolved, and then, it is mixed intimately with near-critical or scCO_2_ by pumping both fluids through a near zero volume tee (<0.1 μL) to generate an emulsion. The emulsion expands through a flow restrictor (50–70 mm long) into a drying chamber at atmospheric pressure to generate aerosols of microbubbles and microdroplets. The tee and the restrictor are heated to a temperature between 50 and 100 °C to avoid the restrictor clogging during expansion [145]. The precipitation chamber contains heated air or nitrogen gas to assist in the drying of aerosol droplets or bubbles. Dry particles are collected on a filter placed at the bottom of the drying chamber [130,139,141,145] (see Figure 8).

This method allows the processing of thermolabile drugs, and it is the preferred method for water-soluble drugs. Organic solvents miscible with the supercritical fluid can be substituted in part or totally for water. Very small particles (<3 µm diameter) can be produced by this method [145].

To make the CAN-BD process more versatile, some modifications have been proposed, including Supercritical Enhanced Atomization (SEA) and Supercritical Fluid Assisted Atomization/Supercritical CO_2_-Assisted Spray Drying (SAA/SASD) [139]. 

##### Supercritical-Enhanced Atomization (SEA)

In this process, the supercritical fluid is used as a nebulization agent. SEA takes advantage of the ability of supercritical fluids to enhance liquid jet dispersion into fine droplets when being depressurized along with liquid solutions. The jet disintegration results in the formation of crystalline sub-micron particles or amorphous materials [139,165]. The SEA process has been extensively used by Padrela et al. in the preparation of cocrystals of different active pharmaceutical ingredients [165,166].

**Figure 9:**
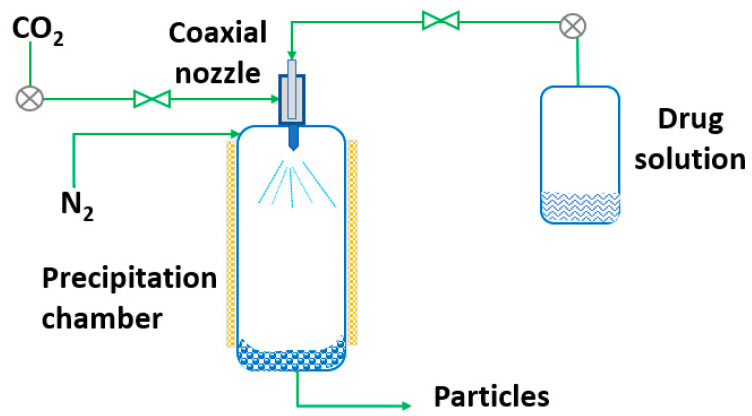
Supercritical-Enhanced Atomization (SEA) process (adapted from Padrela et al. [15]).

The main difference between SEA and CAN-BD is the utilization of a coaxial nozzle with a pre-expansion mixing chamber instead of the micrometric volume tee. The pre-expansion mixing chamber allows the mixing of both fluids at selected conditions of pressure and temperature before its depressurization into a precipitation chamber at atmospheric pressure [139,165,166] (see Figure 9). The SEA process can be easily adapted from existing spray-drying facilities widely used in the pharmaceutical industry. As a disadvantage, it may require higher processing temperatures than CAN-BD [15].

##### Supercritical Fluid-Assisted Atomization (SAA)/Supercritical CO_2_-Assisted Spray-Drying (SASD)

SAA was originally proposed by Reverchon and co-workers in 2002 as a modification of CAN-BD to improve the mixing between CO_2_ and the liquid solution to micronize [167]. In SAA, a thermostatically controlled saturator, packed with stainless steel perforated saddles to ensure good contact between the liquid solution and scCO_2_, is used instead of a low-volume T-join. The liquid solution formed on the contact device is sent to a thin wall injector. Droplets are produced and reach the precipitation chamber where warm N_2_ is supplied for particle formation [130,139,141,168]. The SAA process enhances the efficiency of conventional spray drying, but it presents a better control on particle size distribution and leads to smaller particles (<100 nm) [15].

**Figure 10:**
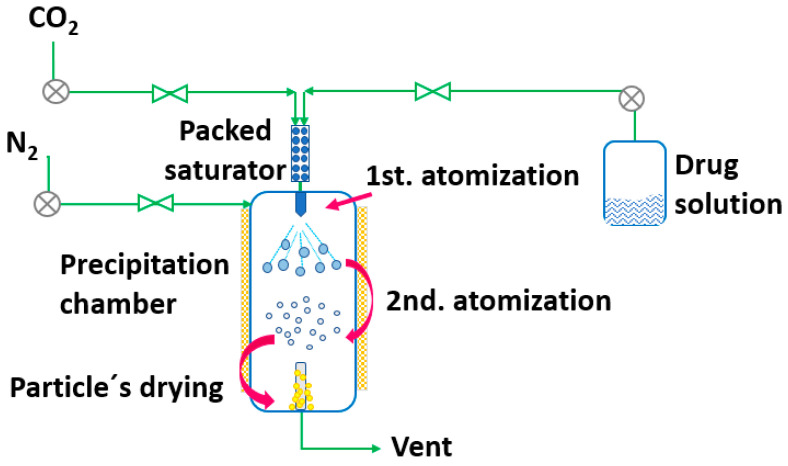
Supercritical Fluid-Assisted Atomization (SAA) process (adapted from Franco et al. [135]).

In SAA, two atomization processes take place: (i) droplets at the nozzle are produced by pneumatic atomization; and (ii) the rapid expansion of scCO_2_ from the primary droplets leads to the jet breakup at the exit of the injector [135,137] (see Figure 10).

The SAA process is widely used in the micronization of pharmaceuticals, catalysts, polymers, and dyes. Water, ethanol, methanol, and acetone are mainly employed, pure or mixed, to process active compounds using this technique [135,141]. The major limitation is the high temperature required for evaporating the liquid solvent, which prevents its application to thermolabile substances [141,169]. Other authors also refer to this process as SASD. SASD facilitates the micronization of hydrophilic and hydrophobic drugs using organic and inorganic solvents, and it is suitable for the production of inhalable composite particles [124,169].

#### 7.1.5. Supercritical CO_2_ as Antisolvent

##### Gaseous Antisolvent (GAS)

**Figure 11:**
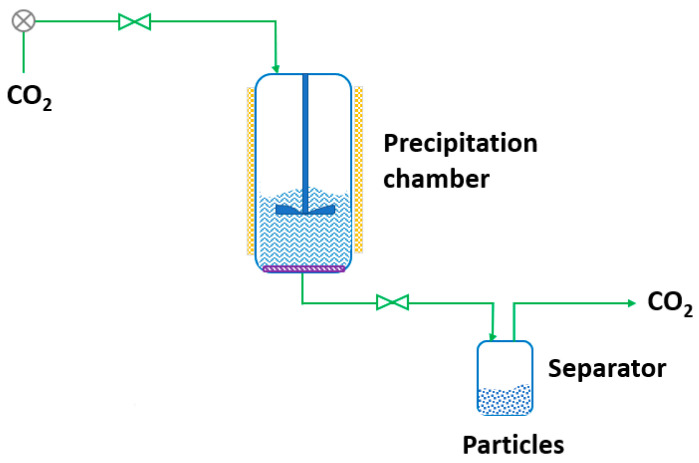
Gaseous antisolvent (GAS) process.

The use of CO_2_ as an antisolvent to crystallize compounds was first proposed in 1989 by Gallagher et al. [170]. This method is used when the solute to micronize is not soluble in CO_2_ but can be dissolved in an organic solvent that is fully miscible with CO_2_. Different organic solvents such as ethanol, methanol, acetone, dimethyl sulfoxide and tetrahydrofuran are currently used [15,16]. In the GAS technique, compressed CO_2_ is added, very often under stirring, to a solute-containing solution in a high-pressure vessel until the desired pressure. The CO_2_ dissolves into the liquid solvent, causing the liquid solvent to expand, its solubilizing power to decrease and the solute precipitates out of the solution [133,145,166] (see Figure 11).

The main advantage of the GAS process over the RESS process Is its versatility, since a wide range of products can be micronized in the range of 1–10 μm, with the only requirement that the solute must be soluble in an organic solvent and insoluble in scCO_2_, which is the most common situation for pharmaceutical compounds. Furthermore, operation pressures are much lower than those used in RESS [123]. 

A disadvantage of GAS is that precipitation takes place under different conditions during the CO_2_ addition, being difficult to assess the effect that each parameter has on the properties of the final product [141,145].

To overcome the limitations, there are several modifications of the GAS process that differ mainly in the way that CO_2_ is mixed with the organic solution, the operation conditions, and the flow regime. These techniques are: Supercritical Antisolvent (SAS), Solution Enhanced Dispersion of Supercritical Fluid (SEDS), Aerosol Solvent Extraction System (ASES), Precipitation with a Compressed Antisolvent (PCA), Supercritical Antisolvent with Enhanced Mass transfer (SAS-EM) and Supercritical Fluid Extraction of Emulsions (SFEE) [133,139,171].

##### Supercritical Antisolvent (SAS)

**Figure 12:**
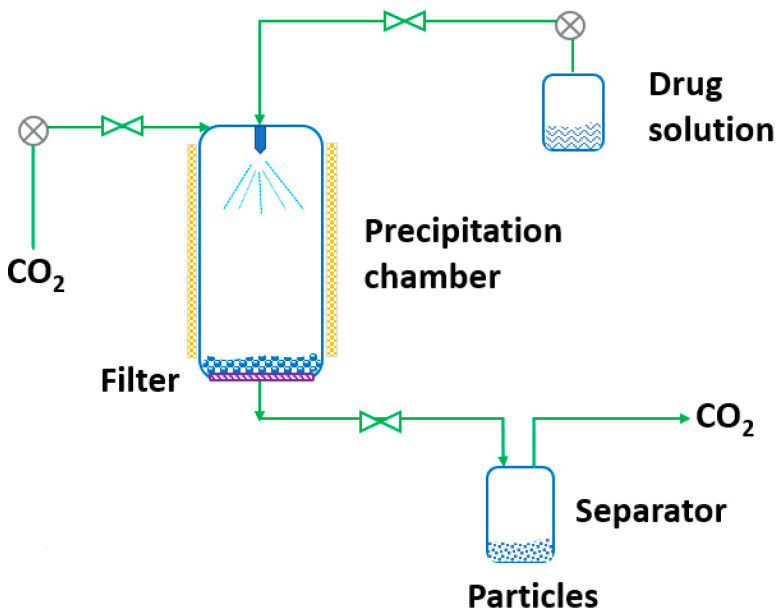
Supercritical Antisolvent (SAS) process (adapted from Martin and Cocero [123]).

In this technique, a solution of the compound to precipitate dissolved in an organic solvent and CO_2_ are continuously pumped into a precipitation chamber filled with scCO_2_ at a fixed temperature and pressure. The solution is injected through a nozzle and forms small droplets. CO_2_ dissolves into the organic solvent, leading to solvent expansion and solution supersaturation and causing the precipitation of the solid in the form of micro and nanoparticles [137,139] (see Figure 12). The micronized particles are collected in the precipitation chamber, which is extensively flushed with pure CO_2_ to remove any solvent residue in the precipitate. The particle size and morphology are controlled by varying the process parameters such as temperature, pressure, solute concentration, flow rates, etc. [172]. Drug amorphization can be achieved by the coprecipitation of the drug with excipients such as PVP [173]. 

This method is widely used to micronize different pharmaceutical substances, catalyst particles, high-energy materials, superconductor precursors, polymers, pigments, and other products which are not soluble in CO_2_. Co-crystals of active pharmaceutical ingredients can be also prepared by SAS [15,166,174,175,176]. Although some SAS micronization processes have been successfully scaled up in pilot plants [141,153], the complex mass transfer processes involved make the implementation at the industrial level difficult [145].

##### Solution-Enhanced Dispersion of Supercritical Fluids (SEDS)

**Figure 13:**
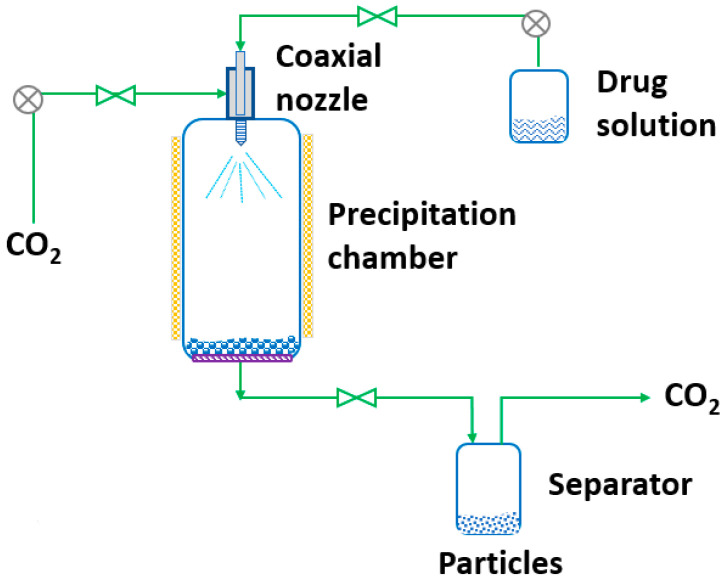
Solution-Enhanced Dispersion by Supercritical Fluids (SEDS) process (adapted from Tran and Park [181]).

This process is a modification of the SAS method, being the main difference in the way that the solution and the antisolvent are contacted. The process was patented by Hanna and York in 1994 [177]. In SEDS, the supercritical fluid and the drug solution are simultaneously introduced into the precipitation chamber at a temperature and particular pressure using a coaxial nozzle, which increases the rate of mass transfer at the interface [135,141,145,178] (see Figure 13). Because of the high mass transfer, the nucleation is fast and results in smaller particle sizes with less agglomeration [15,16]. In SEDS, the particle size depends greatly on the flow rates of scCO_2_ and the active substance solution in the coaxial nozzle. 

This process is also suitable to process water-soluble compounds (e.g., peptides and proteins) using a three-compartment coaxial nozzle by spraying the aqueous solutions along with the organic solvent and CO_2_ separately [179]. The addition of an organic solvent helps to overcome the problems associated with the limited solubility of water in scCO_2_ [130,180]. 

The advantages of this method are related with the improved mass transfer between CO_2_ and the liquid solution which produces small and uniform particles with minimal agglomeration. Furthermore, particles present very low residual solvent content even when the process is operated at reduced drying time and high yield [180,181]. As the SAS technique, the method can be adapted to continuous operations [15]. The main disadvantage of this technique is the easy nozzle blockage [180].

##### Supercritical Antisolvent with Enhanced Mass Transfer (SAS-EM)

This technique was proposed to reduce the size of the particles formed during precipitation and improve the mixing of the SAS method [178]. It was originally developed to create particles for pulmonary delivery [182]. In this process, ultrasound is applied to the injection nozzle, improving the dispersion of the solution into fine droplets [135,141]. The ultrasound field generated by the vibrating surface increases the turbulence and the mixing of the solution and the supercritical antisolvent, altering the particle size and morphology of the particles produced and resulting in a high mass transfer [135,168,182]. The same concept has been applied in SEDS-EM [183].

In comparison to the conventional SAS process, droplet agglomeration is avoided due to the improved and faster mixing, leading to the formation of smaller particles [18,135].

##### Supercritical Fluid Extraction of Emulsions (SFEE)

In SFEE, supercritical CO_2_ is used to rapidly extract the organic phase of an emulsion oil in water (O/W). The compound to be micronized is previously dissolved in this organic phase, which is then emulsified with the aqueous phase. The addition of a small amount of surfactant or emulsifying agents is required. The emulsion is introduced into the precipitation chamber. Then, scCO_2_ is introduced continuously at the bottom, typically at very mild conditions (8.0 MPa and 38 °C). When the solvent is removed, the compound precipitates in small particles that remain suspended in the water. They are stabilized and dispersed in the aqueous phase by the surfactant, preventing aggregation. The process was first patented by Ferro Corporation [184].

**Figure 14:**
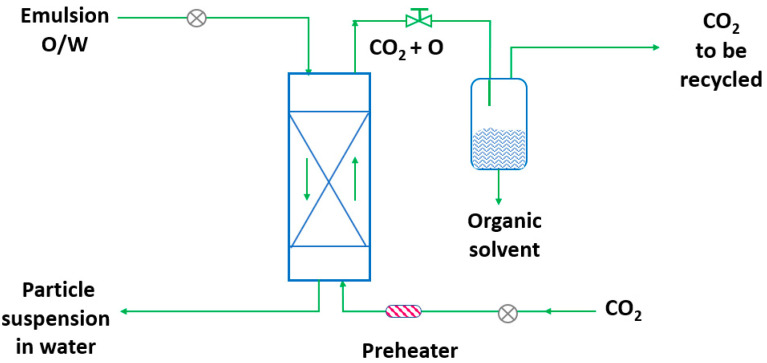
Supercritical Fluid Extraction of Emulsions (SFEE) process (adapted from Prieto et al. [185]).

A variation of the method is used to generate composites. Both the active compound and the support or coating are dissolved in the organic phase; so, when this is removed, homogeneous aggregates or even true core–shell capsules are generated. If the active compound is soluble in water, double emulsions (W/O/W) are used. The SFEE process can be performed continuously in packed columns reducing the CO_2_ consumption and processing time, permitting high production [185]. Both the organic and the supercritical solvent can be recirculated in the process. Figure 14 shows a schematic of the continuous process in a packed column. It has been successfully applied to the production of drug nanoparticles and nanostructured composite particles: for example, for the preparation of bactericidal, anti-inflammatory, or tunable release medications [186,187,188].

This process has a high encapsulation efficiency (>90%) and drug load (>30%), even for liquids, and a very narrow particle size distribution [189,190].

##### Other Supercritical Antisolvent Techniques 

In some publications, the precipitation by compress antisolvent (PCA) technique was proposed [130,178,191]. Depending on the publication, PCA is equivalent to the conventional SAS or SEDS processes. PCA has been efficiently used in the production of a great variety of organic and biopolymer-based particles [145], leading to small solvent-free particles with a narrow size distribution at mild operating conditions [192].

In other publications, the Aerosol Solvent Extraction System (ASES) technique is used. This method was initially developed for the production of polymeric drug delivery systems and has been extended to the encapsulation of various pharmaceutical compounds [15,138]. The ASES process is equivalent to a conventional SAS process. At a high scCO_2_ to solvent ratio, the mass transfer is improved, and fine particles can be obtained [168,178].

#### 7.1.6. Micronization of COVID-19 Drugs

All the COVID-19 drugs considered in this review, except Remdesivir, are administered orally, and most of these drugs have poor water solubility and low bioavailability. The size of the particles, the crystallinity, the shape and the structure determine the absorption and bioavailability of the drugs [168]. Thus, the application of supercritical fluids could significantly improve these attributes.

Table 3 shows the different micronization techniques according to the role of scCO_2_ during the process as well as the drugs from Table 1 and Table 2 and the natural bioactive compounds (Section 4.8) used in the treatment of COVID-19 that have been prepared using these techniques. It also shows the particle size obtained during the micronization process.

The Supercritical Antisolvent techniques are the most frequently applied, as most drugs and bioactives are not very soluble in CO_2_. Then there are a few reports on the use of CO_2_ as a solvent, RESS and its modification, for the micronization of natural materials soluble in CO_2_ and the preparation of drug polymer composites and liposomes. The remaining publications refer to the use of CAN-BD and SAA/SASD.

**Table 3:** Micronization techniques according to supercritical CO_2_ role, applied in drugs or bioactive compounds tested against COVID-19.

scCO_2_ Role	Technique to Micronization Processes		Drugs or Bioactive Compound (Solvent)/Particle Size Obtained
As a solvent	RESS	Rapid Expansion of Supercritical Solutions	Artemisin/0.550–2.100 µm [15,168,193]
			Hesperidin/0.005–0.100 μm [115]
	RESOLV	Rapid Expansion of a Supercritical Solution into a liquid solvent	Melatonine (ethanol) in liposomes/0.066 µm [17,194]
			Artemisin with PVP K25 (dichloromethane)/10.6 ± 0.5 µm [16,145,181,195]
As solute	PGSS	Particles from Gas-Saturated Solutions	Quercetin with HP-β-CD [16]
As cosolute	CAN-BD	Carbon Dioxide-Assisted Nebulization with a Bubble Dryer	Doxycycline (water) [139]
	SAA/SASD	Supercritical Fluid-Assisted Atomization/Supercritical CO_2_-Assisted Spray-Drying	Dexamethasone (acetone, methanol)/0.5–1.2 μm [139,168,196]
As antisolvent	GAS	Gaseous Anti-Solvent	Minocycline (ethanol) [15,197]
	SEDS	Solution-Enhanced Dispersion of Supercritical Fluids	Aescin (ethanol)/0.050 μm [168,198]
			Lutein (acetone, dimethylsulfoxide)/0.20–0.36 µm [16,199,200]
			Emodin with PEG (dichloromethane, methanol)/3–12 μm [199,201]
	ASES	Aerosol Solvent Extraction System	Dexamethasone (dichloromethane, methanol)/<5 μm [16,202] Methylprednisolone (tetrahydrofuran)/~5 μm [171]
	SFEE	Supercritical Fluid Extraction of Emulsions	Quercetin with soy lecithin (ethyl acetate)/0.190 µm [16,135,203]
As antisolvent	SAS	Supercritical Anti-Solvent	Minocycline (ethanol)/0.1–1 µm [16,204,205]
			Azithromycin (ethanol) [181,206]
			Ivermectin with PMMA (acetone)/0.050–0.170 µm [135,199,207]
			Dexamethasone with PVP (ethanol)/1.8–2.5 μm [199,208]
			Quercetin with PVP (dimethylsulfoxide)/0.47–9.52 μm [209]
			Quercetin with CAP (acetone)/0.084–0.145 µm [210]
			Quercetin with EC (ethyl acetate)/0.150–0.350 µm [211]
			Quercetin (ethanol)/0.15–1.24 μm [212]
			Quercetin with poloxamer (acetone)/~1 μm [213]
			Apigenin with HP-β-CD (N,N-dimethyl formamide)/0.392 ± 0.008 μm [199,214]
			Apigenin (dimethylsulfoxide)/0.400–0.800 µm [135,168,215]
			Lutein with PLA (ethyl acetate)/1–5 μm [199,216]
			Lutein with PEG (dichloromethane) /5–10 μm [199,217]
			Hesperidin with stearic acid and tween 80 (dimethylsulfoxide)/0.152–0.267 μm [135,199,218]
	SAS-EM	Supercritical Anti-Solvent with Enhanced Mass Transfer	Quercetin (ethanol)/0.120–0.450 µm [168,219]

The micronization of azithromycin by SAS has been successful performed, leading to an extraordinary increase in the solubility and dissolution rate due to the formation of very fine and spherical nanoparticles [181]. A solution of azithromycin in ethanol with PEG 6000 and sodium lauryl sulfate as surfactants were used. The increased dissolution rate was due to the amorphization of the drug during the micronization process [206].

Sievers et al. have micronized the antibiotic doxycycline using water as a solvent by the CAN-BD technique to prepare an inhalable drug [139]. Similarly, corticosteroid dexamethasone was micronized by the ASES technique, aiming at their pulmonary administration, using dichloromethane and methanol as solvents. The mean particle size obtained was lower than 5 µm [16,202]. SAA has been also applied to methanol and acetone solutions of the same drug. Spherical particles with a size of 0.5–1.2 μm were obtained. The resulting particles significantly enhanced the inhibition of tumor necrosis factor in vitro [139,168,196]. In another work, SAS precipitation was proposed for the coprecipitation of dexamethasone in ethanol with polyvinylpyrrolidone (PVP). The particles’ mean diameters ranged from 1.8 to 2.5 μm. The dissolution rate of the composite in phosphate-buffered saline solution (PBS) was more than four times faster than that of the unprocessed drugs [199,208].

Another antibiotic in which micronization techniques have been applied using supercritical fluids is minocycline. Rodrigues et al. demonstrated the conversion of a hydrated form of commercial minocycline hydrochloride to an anhydrate form using the GAS method with ethanol as solvent, removing water molecules from the crystal structure of the drug and generating a new solid-state form [15,197]. Cardoso et al. applied the SAS technique to this same drug. Minocycline was precipitated in a continuous mode from an ethanol solution, yielding relatively stable amorphous particles with almost twice the density of the starting material, with a size ranging from 0.1 to 1 µm, depending on the operating conditions [16,204,205].

Ivermectin has been encapsulated in nanoparticles of poly (methyl methacrylate) (PMMA) with a mean size of 0.050–0.170 µm. A continuous SAS process using acetone as the organic solvent was used to obtain a formulation for a controlled release. In this case and due to the characteristics of the drug evaluated, the researchers recommend the administration of these nanoparticles through the intravenous route [135,199,207].

The ASES technique has been applied to the micronization of methylprednisolone (group of corticosteroids), using tetrahydrofuran as solvent. Micronization led to particles of size close to 5 μm, which is suitable for pulmonary administration [171].

Melatonin, an alternative drug in the treatment of COVID-19, has been encapsulated in liposomes with phosphatidylcholine and cholesterol, using the RESOLV. The mixture was expanded over an ethanol solution. Round or oval particles with an average size of 0.066 μm and uniform particle size distribution were obtained, improving their characteristics for oral administration [17,194].

In addition to the micronization experiments indicated in Table 3, other formulations of the drug niclosamide have been prepared using scCO_2_. This compound has been impregnated onto a silica aerogel by adsorption from a solution including this compound dissolved in scCO_2_. The compound adsorbed on the support is then released in a controlled way and can be used as a drug delivery system [16,220].

Complementary to the indicated drugs, in some natural compounds, micronization techniques with supercritical fluids have also been used, as is the case of quercetin, which is a flavonoid considered one of the most promising candidates in the fight against COVID-19. Several micronization techniques such as SFEE, SAS, SAS-EM and PGSS have been applied to quercetin. 

The SFEE of quercetin with soy lecithin as a coating material an ethyl acetate and water as solvents led to particles of mean particle size equal to 0.190 μm and the complete encapsulation of quercetin in an amorphous state without the presence of segregated crystals [16,135,203]. 

Quercetin has been also micronized by SAS and several of its variants [135,199]. Ozkan et al. reported the SAS precipitation of quercetin using dimethyl sulfoxide as solvent and PVP as the polymeric carrier. Spherical microparticles with mean diameters between 0.47 and 9.52 μm were obtained [209]. The entrapment efficiency in PVP was 99.8% and the dissolution rate from the coprecipitated powder was 10 times faster compared to the dissolution rates of unprocessed quercetin. García et al. have also used SAS to precipitate microparticles of quercetin with cellulose acetate phthalate (CAP) and acetone. Spherical particles with a range between 0.084 and 0.145 μm of diameter were obtained. Release profile studies showed a faster release and higher solubilities of quercetin [210]. Fernández et al. studied the coprecipitation of quercetin particles with the polymer ethyl cellulose (EC) with ethyl acetate as solvent. In this case, amorphous particles in the submicron range with sizes between 0.150 and 0.350 µm were formed. The precipitate characteristics and the high encapsulation efficiency would certainly improve its oral administration route [211]. Montes et al. carried out comparative studies of the SAS micronization of quercetin using ethanol as solvent to evaluate the factors that determine a better particle size distribution. The precipitates were crystalline with a particle size range of 0.15–1.24 μm and presented needle-shape particles forming aggregates [212]. Fraile et al. encapsulated quercetin in Pluronic F127 poloxamer using acetone as a solvent using SAS. Their results indicated a significant reduction in particle size (≈1 μm), the absence of segregated crystalline particles, and an improvement in the dissolution rate [213].

The application of the SAS-EM technique on quercetin was studied by Kakran et al. using ethanol as a solvent. Particles between 0.120 and 0.450 μm were obtained. The dissolution rate of the precipitated material increased significantly in comparison with the original powder [168,219]. Finally, the PGSS technique was applied to quercetin in an inclusion complex with hydroxypropyl-β-cyclodextrin (HP-β-CD) to improve its solubility and dissolution rate and enhance its bioavailability [16].

Other bioactive components have been also micronized using supercritical fluids, Huang et al. have prepared an inclusion complex of apigenin with HP-β-CD using SAS with N,N-dimethylformamide as the solvent with a mean particle size of 0.392 ± 0.008 µm. Significant improvements in the solubility, the dissolution rate and the bioavailability of the bioactive compound were obtained in comparison to the original component [199,214]. Zhang et al. have also prepared nanocrystals of apigenin using the SAS technique with dimethylsulfoxide as a solvent. Spherical nanocrystals with particle sizes between 0.400 and 0.800 µm were obtained. The nanocrystals exhibited faster dissolution profiles than the original powder [135,168,215].

The SEDS technique has been applied to aescin by Jia et al., using ethanol as the solvent, resulting in the production of spherical-shaped nanoparticles with an average diameter of 0.050 μm, and prospective potential for use in oral drug delivery with reduced side effects [168,198]. 

The RESS technique has been applied to artemisinin, another bioactive compound, by Yu et al., who obtained lamella microparticles with a size of 0.550–2.100 μm. The precipitate had lower crystallinity than the starting material and great potential in drug delivery systems [15,168,193]. Van Nijlen et al. have applied the same technique but precipitated this compound over dichloromethane containing PVP K25 (RESSOLV). The polymer acted as a carrier, improving the dissolution rate of the compound in comparison with the starting material. The median particle diameter obtained was 10.6 ± 0.5 µm for oral delivery, and the dissolution rate of the micronized forms was improved in comparison to the untreated form [16,145,181,195].

The SAS process has been used by Miguel et al. to re-crystallize lutein from ethyl acetate solutions [199,216]. As a result of the precipitation, the purity of the lutein increased from 75% to over 90%. They also coprecipitated lutein and poly-lactic acid (PLA). It was possible to reduce the particle size, obtaining spherical particles with a diameter between 1 and 5 μm. Martin et al. have also applied the SAS technique to the precipitation of lutein with polyethylene glycol (PEG) and dichloromethane as a solvent. The size obtained varied between 5 and 10 μm [199,217]. Additionally, Hu et al. have studied the application of the SEDS technique for the production of lutein nanoparticles using acetone and dimethylsulfoxide as solvents. Sub-microparticles with a mean size of 0.20–0.36 µm and controlled release capability were obtained [16,199,200].

The SEDS process with a prefilming twin-fluid atomizer, using PEG and a mixture of dichloromethane–methanol has been applied on emodin by Lang et al. to obtain composite microparticles with a mean size between 3 and 12 μm [199,201]. 

Finally, hesperidin has been loaded in solid lipid nanoparticles by Saad et al. using SAS to improve its oral delivery. Hesperidin was mixed with stearic acid and tween 80, as a surfactant, and it was dissolved in dimethylsulfoxide. The micronization of hesperidin improves its solubility, apparent permeability, stability and bioavailability. The particle size obtained was 0.152–0.267 µm [135,199,218]. Salehi et al. have studied the micronization and coating of hesperidin and hesperitin using RESS; in this case, spherical nanoparticles with a mean size of 0.005 to 0.100 μm were coated [115]. The solubility rate and antioxidant activity of the nanoparticles formed in an aqueous medium increased significantly compared to the original form, demonstrating an improvement in the oral bioavailability of the flavonoids.

Overall, there is a clear potential use of supercritical fluids on the micronization of drugs to enhance their oral and pulmonary availability.

### 7.2. Extraction of Natural Compounds by Supercritical Fluids

The ability of compressed fluids (pressurized liquids) and supercritical fluids to extract targeted compounds highlights them as powerful green techniques with demonstrated capacity to obtain bioactive compounds with antiviral and anti-inflammatory activity [14] applied in different respiratory diseases [109]. 

The main technologies available include the extraction with supercritical carbon dioxide (SFE), liquids under pressure (PLE), subcritical water (SWE), and the use of gas expanded liquids (GXL) [221]. By changing the solvents, the extraction methods, and the conditions, it is possible to tune the target extracts.

Concerning the anti-inflammatory activity, some of the component extracts obtained with supercritical fluids contain flavonoids that have been successfully tested against respiratory viruses, and so, they could be also tested to fight SARS-CoV-2 [109]. For example, Salehi et al. have pursued the extraction with CO_2_ of the hesperidin and hesperitin from sweet orange peels [115], while Kawamoto et al. did it from Citrus genkou [222,223]. Similarly, Nuralin et al. have evaluated the extraction of flavonoids from *Pinus brutia* [224]. 

The SWE technique has been applied in *Citrus unshiu* peel extracts and their acid hydrolysates to evaluate the antioxidant and in vitro anti-inflammatory activities of flavonoid compounds such as hesperidin. SWE extract showed a higher anti-inflammatory response compared to hot water and ethanol extractions [14,225].

Nieto et al. used PLE for the production of extracts from *Vitis vinifera* L. stem and seed with significant anti-inflammatory activity, due to phenolic compounds, including quercetin [14,226]. This compound has been also found in the ethanol-modified supercritical CO_2_ extracts from *Leptocarpha rivularis* stalks [14,227].

Furthermore, the high anti-inflammatory and antioxidant activity of the spinach leaves extracts obtained by SFE and PLE containing carotenoids, such as lutein, was confirmed. In vitro studies demonstrated that the SFE extract showed higher activity than the PLE extract [14,228].

On the other hand, some natural plant extracts obtained with compressed fluids have also proven antiviral activity for respiratory infections. For example, the subcritical water extract of *Brassica juncea L.* showed antiviral effects against influenza virus A/H1N1 [229]. The supercritical CO_2_ extracts from *Cinnamomum cassia L.* ramulus had strong inhibition efficacy against the respiratory syncytial virus [230]. In addition, the extract of *Centipeda minima L*. (a Chineses medicinal herb) possessed good in vitro activity against influenza. The responsible components of this activity were identified as pseudoguaianolides [231]. The edible and medical pericarps of *Citrus reticulata* ’Chachi’ were extracted with supercritical CO_2_, and up to five active polymethoxylated flavones in the extracts were effective in fighting the respiratory syncytial virus [232].

Finally, other components have also been obtained by supercritical extraction, such as terpenoid artemisinin from *Artemisia annua L*. exhibiting antifibrotic activity [223,233].

Table 4 shows a list of natural species on which supercritical fluid extraction techniques have been applied, indicating the composition of the extracts obtained and their action in respiratory diseases.

**Table 4:** Some natural components obtained with compressed fluids and their possible activity against respiratory diseases.

Technique	Raw Material	Extract Components	Tested Activity (Virus Type)
SFE	*Artemisia annua* L.	Artemisinin, artemisin and dehydroartemisinin	Antifibrotic [223,233]
SWE	*Brassica juncea* L.	Glucosinolates, phenolic compounds, flavonoids, phytic acid and brassinosteroids	Antiviral (Influenza virus A/H1N1) [229]
SFE	*Centipeda minima* L.	Sesquiterpene lactones, pseudoguaianolides (Brevilina A)	Antiviral (Influenza virus A/Puerto Rico/8/34 H1N1) [231]
SFE	*Cinnamomum cassia* L.	Cinnamaldehyde, 4-Methoxycinnamaldehyde	Antiviral (Respiratory syncytial virus, Influenza virus) [230]
SFE	Citrus genkou	Flavonoids: hesperidin, nobiletin and tangeretin	Antioxidant and anti-inflammatory [222,223]
SFE	*Citrus reticulata*	Flavonoids (tangeretin and nobiletin)	Antiviral (Respiratory syncytial virus) [232]
SFE	*Citrus sinensis* L.	Bioflavonoids: Hesperidin and hesperitin	Antioxidant and anti-inflammatory [115]
SWE	*Citrus unshiu*	Flavonoids: hesperidin	Antioxidant and anti-inflammatory [14,225]
SFE	*Leptocarpha rivularis*	Caryophyllene oxide, quercetin, kaempferol and resveratrol	Antioxidant and anti-inflammatory [227]
SFE	*Pinus brutia*	Terpenoids, flavonoids (Quercetin, rutin, kaempferol), lignands, polyphenolics and oily content	Antioxidant and anti-inflammatory [224]
PLE	*Vitis vinifera* L.	Proantocyanidins (transresveratrol) and quercetin derivatives	Anti-inflammatory [226]

### 7.3. Virus Inactivation and Sterilization of Contaminated Material

The supercritical processes are inherently sterile and can be completed in a single stage, in closed high-pressure stainless-steel systems, with few moving parts, avoiding too much exposure of the products to external environments [15,145]. With a proper cleaning program, cross-contamination can be avoided.

Moreover, some compressed fluids have proven to have high antimicrobial and antiviral capacity when in contact with food, tissues, polymers, and materials used in the manufacture of medical devices, implants and biotissues. Thus, it has been proposed as an alternative technique to radiation, autoclaving and oxidizing gases, which can cause damage to hydrolytic and thermosensitive materials, avoiding their reuse [234].

For example, the efficacy of scCO_2_ treatment has been demonstrated in different virus families. The sterilization of HIV1, Sindbis, Polio Sabin I and Pseudorabies on bone grafts has been achieved on a pilot scale [235,236] and that of Sindbis, Parainfluenza, PHV1, Vaccinia, HIV1 injected into plasma products at temperatures of less than 32 °C in minutes [237]. It even led to the destruction of more than six orders of magnitude of coronaviruses at 40 °C and 16 MPa in 30 min has been described [237]. The sterilization conditions of porcine encephalomyocarditis virus were 35 °C, 10 MPa for 15 min, when 55 ppm peracetic acid was added to CO_2_ [238]. Various bacteriophages have been also removed with CO_2_ at 8.5 MPa and 38 °C, modified with water (0.25%) + H_2_O_2_ (0.15%) + acetic anhydride (0.50%) [239]. 

Table 5 lists the published work on the virucidal activity of supercritical CO_2_ and that on materials involved in the COVID-19 crisis.

**Table 5:** Virucidal activity of supercritical CO_2_ and that on materials involved in the COVID-19 crisis.

Virus	Medium	CO_2_ Application Conditions				Reduction
		Pressure (MPa)	Temperature (ºC)	Time (min)	Additives	(log^10^)
HIV-1	Allografts	25	50	10 with continuous flow of CO_2_	None	>4.0
Sindbis (Hepatitis C model)		25	50			>4.3
Polio Sabin (Hepatitis A model)		25	50			>6.6
Pseudorabies (Hepatitis B model) [235,236]		25	50			>4.0
Sindbis	Plasma	25	32	150	None	6
Parainfluenza type 3		25	32			6.1
Porcine Herpesvirus 1 (PHV-1)		25	32			5.5
Vaccinia		25	32			6.2
HIV-1 [237]		25	32	30		5
Coronavirus		16	40	30	None	>6.0
Herpesvirus		16	40			>7.0
Flavivirus		16	40			>8.0
Arterivirus [240]		16	50			>9.0
Porcine encephalomyocarditis [238]	Porcine acellular matrix	9.4–10	35–41	15 with agitation	Peracetic acid (55 ppm)	>6.4
Bacteriophage MS2	Resorbable 3D implant models	8.5	38	5	Water (0.25%) + H_2_O_2_ (0.15%) + acetic anhydride (0.5%)	>6
Bacteriophage Phi X 174 [239]						>6
HCoV-NL63	Surgical, cloth masks and N95 respirators	10.3–13.8	33–35	5–90	None	---
SARS-CoV-2 coronavirus [241]						
---	3M 1860 N95 masks [242]	8.3	37	60	None	---
---	Filtering facepiece respirators FFP2 [243]	7.5	70	60	Ethanol and hydrogen peroxide (30%)	---

N_2_O has been also tested at a pressure of 25 MPa and temperature between 37 and 50 °C for 2 h for the inactivation of Sindbis, BVDV, PRV, Polio 1 and Reo 3. It has shown to be more efficient for enveloped viruses, which is probably due to the extraction of the lipids of this envelope [234].

A patent covers the antiviral capacity of several fluorocarbons (R22, RTR134a, R124, R23) applied at 50 °C and 21 MPa [244].

Some recent papers prove the suppression of SARS-CoV-2 virus in different types of biological protective masks to explore the possibility of reusing them, given their scarcity in the COVID-19 pandemic.

One research has looked at surgical, cloth masks and N95 respirators, demonstrating the complete inactivation of HCoV-NL63 and SARS-CoV-2 coronavirus at 10.3–13.8 MPa and 33–35 °C from 5 to 90 min. It also identified different mechanisms of viral inactivation in scCO_2_ sanitization, such as membrane damage, germination defect and dipicolinic acid leakage. In addition, no changes in the morphology, topographical structure or integrity of the evaluated kits were detected. Most importantly, the post-processing wettability was preserved [241]. 

Similarly, a SANDIA report released in 2020 describes the findings on the CO_2_ treatment of 3M 1860 N95 masks. A sample of fifteen of these masks was exposed to ten consecutive cleaning cycles at 37 °C and 8.3 MPa for 1 h. They were then subjected to a series of standardized quality tests comparing the results with five control samples. These tests covered pressure/flow characteristics, particle filtration efficiency and proper fit of the masks. The results suggested (but did not prove) that supercritical carbon dioxide does not damage 3M 1860 N95 masks. Further testing confirmed the compatibility of supercritical CO_2_ with the ventilator tubing that has been in such short supply during the COVID-19 pandemic [242].

Another study has evaluated the treatment of filtering facepiece respirators FFP2 with scCO_2_ with a cleaning solution, containing ethanol and hydrogen peroxide 30%. The tests were performed for 1 h at 7.5 MPa and 70 °C. A biological indicator was used to test the sterilization efficiency. Then, the washing of organic deposits and the maintenance of the filtration performance was evaluated. It was possible to demonstrate compliance with the requirements for their safe reuse [243].

## 8. Conclusions

To date, there are no fully effective therapies to fight COVID-19 and avoid its transmission. The development of new drugs is a very long process of high risk, and there is no guarantee of success in the short term. Therefore, the repositioning of available antivirals and other already known drugs, alone or in combination, appears as a promising strategy to combat COVID-19. The fact that the pharmacological, pharmacokinetic, and toxicological profiles of the drugs are already known makes the process faster. 

The medicines that are currently used to treat COVID-19 and their secondary complications are mainly antivirals, corticosteroids, anti-infective, antiparasitic, antineoplastic and immunomodulating agents. Many of these medicines are poorly soluble in physiological fluids and are badly absorbed in the gastrointestinal tract, negatively affecting their bioavailability. Much research is therefore being completed on strategies to improve this aspect, including their inclusion in carriers and their size reduction. In both cases, CO_2_-based micronization techniques can provide many advantages, since it is possible to prepare composites with different polymers, ceramics and liposomes and at the same time finely control the drug particles size in the range of interest for each application, from the micron to the nanoscale, which is difficult to achieve by conventional drug manufacturing techniques.

Since the respiratory tract is the route of entry for the COVID-19 disease, inhalation administration should be considered the best option for the treatment of COVID-19, complementing oral administration. For that, particle size should be in the 1–5 micron range. Such a size range can be achieved with CO_2_-based precipitation techniques with a narrow particle size distribution. Using supercritical fluids to optimize particle characteristics may improve the drug bioavailability and allow reducing their dose and consequently, their adverse effects. The pharmaceutical industry is exploring these techniques, mainly in pilot-scale plants. On the other hand, in academia, there is a lot of room for research into the preparation of antivirals for both oral and inhalation administration.

Bioactive compounds obtained from plants with antiviral and anti-inflammatory activity to work against COVID-19 have been also proposed. Their use in combination with some of the previous medicines may help to alleviate the symptoms and possible complications derived from this disease. The application of supercritical fluids for their extraction and micronization may provide numerous benefits compared to traditional methods, as evidenced by the numerous studies carried out to date. 

CO_2_ and some other supercritical fluids have demonstrated their antimicrobial capacity in many media. The application of supercritical fluids in the inactivation of viruses and personal protective equipment, as well as in tissues, scaffolds, allografts, and blood plasma (biological fluid) is an excellent alternative to traditional techniques. There are already companies selling specialized equipment, but there is a lack of regulatory development for its use as a sterilization technique. We foresee that given its effectiveness, this technique will eventually consolidate.

The development of all these technologies is also intended to enable society to take advantage of all the benefits of COVID-19 research to anticipate other potential pandemics that encompass the ONE-HEALTH concept and thus have tools for future containment.
